# Multi-Level Kinetic Model of mRNA Delivery via Transfection of Lipoplexes

**DOI:** 10.1371/journal.pone.0107148

**Published:** 2014-09-19

**Authors:** Thomas S. Ligon, Carolin Leonhardt, Joachim O. Rädler

**Affiliations:** Faculty of Physics and Center for NanoScience (CeNS), Ludwig-Maximilians-Universität, München, Germany; Centrum Wiskunde & Informatica (CWI) & Netherlands Institute for Systems Biology, Netherlands

## Abstract

Recent work on the use of mRNA lipoplexes for gene delivery demonstrates the need for a mathematical model that simulates and predicts kinetics and transfection efficiency. The small copy numbers involved make it necessary to use stochastic models and include statistical analysis of the variation observed in the experimental data. The modeling requirements are further complicated by the multi-level nature of the problem, where mRNA molecules are contained in lipoplexes, which are in turn contained in endosomes, where each of these entities displays a behavior of its own. We have created a mathematical model that reproduces both the time courses and the statistical variance observed in recent experiments using single-cell tracking of GFP expression after transfection. By applying a few key simplifications and assumptions, we have limited the number of free parameters to five, which we optimize to match five experimental determinants by means of a simulated annealing algorithm. The models demonstrate the need for modeling of nested species in order to reproduce the shape of the dose-response and expression-level curves.

## Introduction

Quantitative analysis of transfection is important for gene therapy involving plasmid DNA and mRNA, as well as high-throughput screening (HTS) and siRNA research [1]–[4]. For this reason, it is important to know more about the kinetics and dose-response relationship for delivery of genes and RNA-based nucleic acid constructs and to understand the common principles that underlie nucleic acid pharmacokinetics in any given cell type. Many studies have collected quantitative data on the uptake and pathway of gene carriers [5]–[10] and the physico-chemical characterization of cationic lipoplexes and polyplexes has been reviewed extensively [11]–[17]. In the last few years, first theoretical considerations modeling the uptake and pharmacokinetics of lipolexes using biochemical reaction kinetics have been undertaken [18]–[20]. Some specialized models also address the spatial distribution and active transport along microtubules [21]. The stochastic nature of in the delivery process has been investigated for nanoparticles [22] and for plasmid DNA [23]. The use of movies for the analysis of single-cell tracking experiments has been reviewed [24]. For modelling of biological systems in general, there is an emerging set of tools in the context of systems biology, including a new generation of computational methods, such as process calculi and “executable biology” [25]. In fact, many biological reactions require addition of stochastic modeling as well as spatial aspects that go beyond reaction and diffusion [26]. For example, endosomes contain lipoplexes and lipoplexes contain mRNA molecules, and this can lead to a combinatorial explosion in the number of variables and equations. The transfection process requires the use of modeling techniques that have not been used often, because substances can be contained in each other.

The problem of multi-level modeling has been treated in many investigations and tools. Systems Biology Markup Language (SBML) [27] and tools based on it, for example Cell Designer [28] and Copasi [29], include the concept of compartments, which contain species, but the compartments are only containers that cannot support reactions of their own. First attempts to allow modelling with compartments include the process calculus Pi Calculus [30]–[35] and tools based on it, such as BioAmbients [36], Beta-Binders [37]–[39] and the Stochastic Pi Machine SPiM [40]. In addition, the “rules-based” language BioNetGen Language BNGL [41] and tools based on it, such as NFsim [42], contain some very explicit methods for handling nested structures. One example where these techniques were used is a model for the uptake of nanoparticles is the work by Dobay et al. using SPiM [43], which also demonstrates the need for multi-level modeling in many situations involving nanoparticles.

Recently, we showed that quantitative analysis of transfection at the single-cell level makes it possible to analyze the stochastic aspects of transfection quantitatively [23], [44]. The single cell exhibits time courses that are characterized by a distinct delay time before the onset of expression, a phase of GFP increase and finally a steady state level. We showed that the distribution of steady-state levels was related to the number of successfully delivered plasmids and well described by an analytical model [23]. In the same spirit, we analyzed the transfection of mRNA, which is more homogeneous and earlier compared to pDNA [45], [46]. However, there is yet little understanding regarding the kinetics of mRNA delivery. It is generally accepted that mRNA lipoplexes are taken up via clathrin-dependent endocytosis [47]. Existing models for RNA delivery sometimes include a single “internalization” reaction, but that is not sufficient for reproducing the data created by single-cell tracking experiments. In particular, there is no kinetic model for the delivery of mRNA that explicitly takes the compartments of the transfer process into account.

Here we present a mathematical model, based on mass-action kinetics, which describes the uptake of mRNA lipoplexes via endocytosis and endosomal lysis. Our goal was to create a kinetic model that reproduces experimental data, especially the distribution of time courses, and supports predictive modeling. While the investigation of plasmid DNA [23] provides some background and motivation, this model was based solely on the data published on the experiment with mRNA [44]. We demonstrate that the uptake kinetics is well described by a stochastic, mass action based model that accounts for uptake of multiple lipoplexes. We solve the problem of parameter estimation by choosing well-known rate constants from literature and keeping five kinetic rates free, which we optimize to meet the constraints of the experimental transfection statistics and measured onset time distribution by using a simulated annealing algorithm. As such, the model yields uptake behavior that reproduces the experimental data and is capable of predicting behavior beyond the experimental parameter regimes. The model also demonstrates the need for modeling of nested species as well as modeling kinetic reactions in a stochastic version in order to reproduce the shape of the dose-response and expression-level curves, and the need to include the maturation step in order to reproduce the variance of the onset-time distribution. The benefit of predictive modeling and the known limitations of the model are discussed.

## Model Description

### Streamlined Model

**Figure 1:**
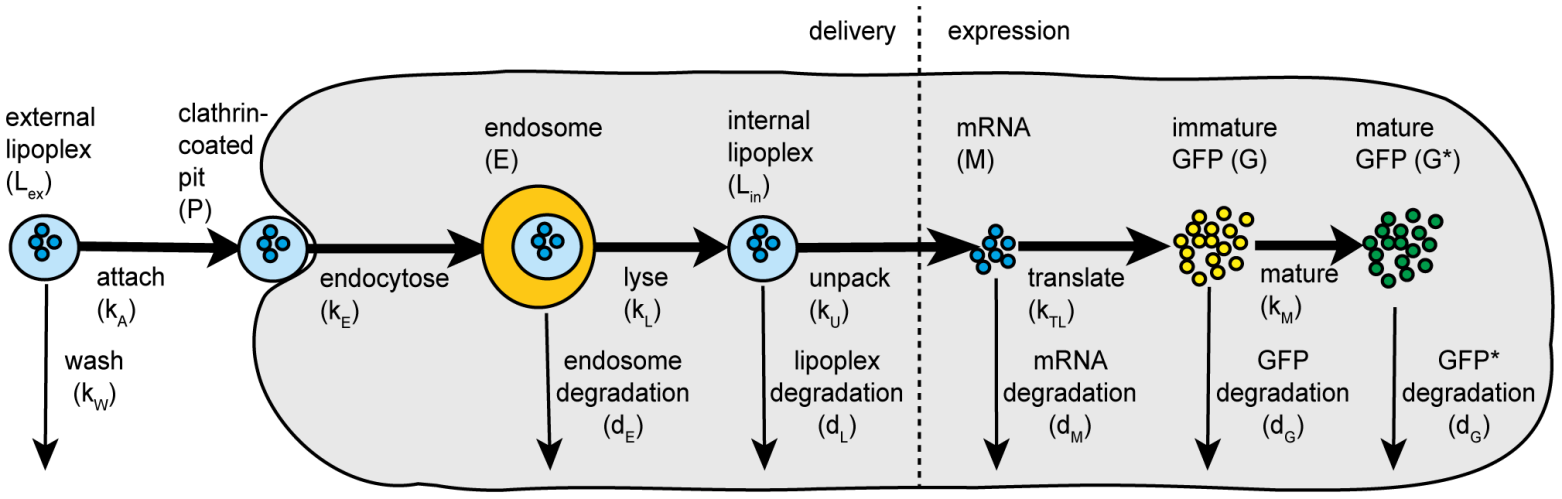
Diagram of the streamlined transfection model. External (extracellular) lipoplexes attach to the surface of the cell, forming clathrin-coated pits, which enter the cell via endocytosis, leading to the formation of endosomes, which either lyse or degrade. This puts the lipoplexes into the cytosol, where they unpack, releasing the mRNA, which translates to unfolded GFP molecules, which then mature (folding and oxidation), to produce active GFP. In addition to the endosomes, the lipoplexes, mRNA, immature and mature GFP are all degraded at set rates.

We model mRNA transfection by a sequence of mass-action type chemical reactions (shown in Figure 1), which can be divided into the delivery of lipoplexes and the GFP expression via the mRNA released.

The delivery phase is described by the following ODEs:
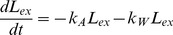
(1)

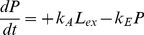
(2)

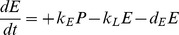
(3)

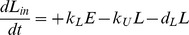
(4)

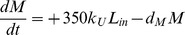
(5)


Where *L_ex_* is the concentration of external lipoplexes, *k_A_* is the rate at which lipoplexes attach to the cell surface, *k_W_* is the washing rate, which is equal to zero at first and jumps to a high value after the incubation time or normally one hour, *P* is the concentration of clathrin-coated pits (i.e. number per cell), *k_E_* is the rate of endocytosis, *E* is the concentration of endosomes (i.e. number per cell), *k_L_* is the rate of lysis of endosomes, *d_E_* is the rate of endosome degradation, *L_in_* is the concentration of internal lipoplexes, *k_U_* is the rate of lipoplex unpacking, *d_L_* is the rate of degradation of lipoplexes, *M* is the concentration of mRNA, *k_U_* is the rate of unpacking of lipoplexes, and *d_M_* is the rate of degradation of mRNA. The degradation of endosomes is primarily a model parameter to represent endosomes that are never observed to lyse, and includes mRNA degradation in the endosome.

The expression phase is described by the following ODEs, plus equation (5), which includes mRNA degradation:
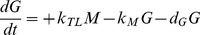
(6)

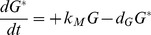
(7)


Where *G* is the concentration of immature (unfolded) GFP, *k_TL_* is the rate of translation, *k_M_* is the rate of maturation (folding and oxidation), *d_G_* is the rate of degradation of both immature and mature GFP, and G* is the concentration of mature GFP. The reaction rates are documented in Table 1.

**Table 1:** Rates.

A parameters, fitted (optimized) and fixed						
	role	goal (exp.)	streamlined with slow maturation	multiple-lipoplex with fast maturation	multiple-lipoplex with slow maturation	literature
k_A_ (attach)	fitted		.03	0.26	0.27	0.006–0.5 [20], [21], [59]
k_E_ (endocytosis)	fitted		.8	0.73	0.81	0.16–0.5 [20], [21], [59]
k_L_ (lysis)	fitted		.065	0.10	0.11	0.001–0.96 [20], [21], [59]
k_M_ (maturation)	fitted or fixed		5.5	9.23	5.5	0.5–9.23 [48], [60]–[63]
d_E_ (endosome degradation)	fitted		0.65	0.60	0.67	n.a.
k_U_ (unpack)	fixed		1e+06	1e+06	1e+06	n.a.
d_L_ (lipoplex degradation)	fixed		1e–06	1e–06	1e–06	n.a.
k_TL_ (translation)	fixed		170	170	170	170 [44]
d_M_ (mRNA degradation)	fixed		0.062	0.062	0.062	0.062 [44]
d_G_ (GFP degradation)	fixed		0.056	0.056	0.056	0.056 [44]

B experiment vs. simulation						
TE (transfection efficiency)	target	40	44	36	38	
LC (lipoplexes on cell)	target	6	6.43	6.02	6.03	
maxGFP	target	7.09e+5	4.32e+5	4.91e+5	5.34e+5	
t0-mean	target	3.14	3.36	3.49	3.23	
t0-width	target	1.54	1.72	2.05	1.65	

A) The table shows the rate constants used by the simulation. During optimization, k_A_, k_E_, k_L_, and d_E_ were varied, and k_M_ was varied in one case. Column “streamlined with slow maturation” is the streamlined model with k_M_ = 5.5 fixed. Column “multiple-lipoplex with fast maturation” is the multiple-lipoplex model with k_M_ = 9.23 fixed to the value from literature. Column “multiple-lipoplex with slow maturation” is the multiple-lipoplex model with k_M_ varied (optimized). The literature values are described in more detail in the File S1. B) The last 5 rows are the experimental data used as a goal in optimization.

This first model shows a very linear progression of single lipoplexes attaching to and entering the cell, but we know from experiment that endosomes can contain multiple lipoplexes, so we need to address that and allow for endocytosis of multiple lipoplexes per endosome. This means that we will have multiple levels of containment.

### Multi-Level Modeling

**Figure 2:**
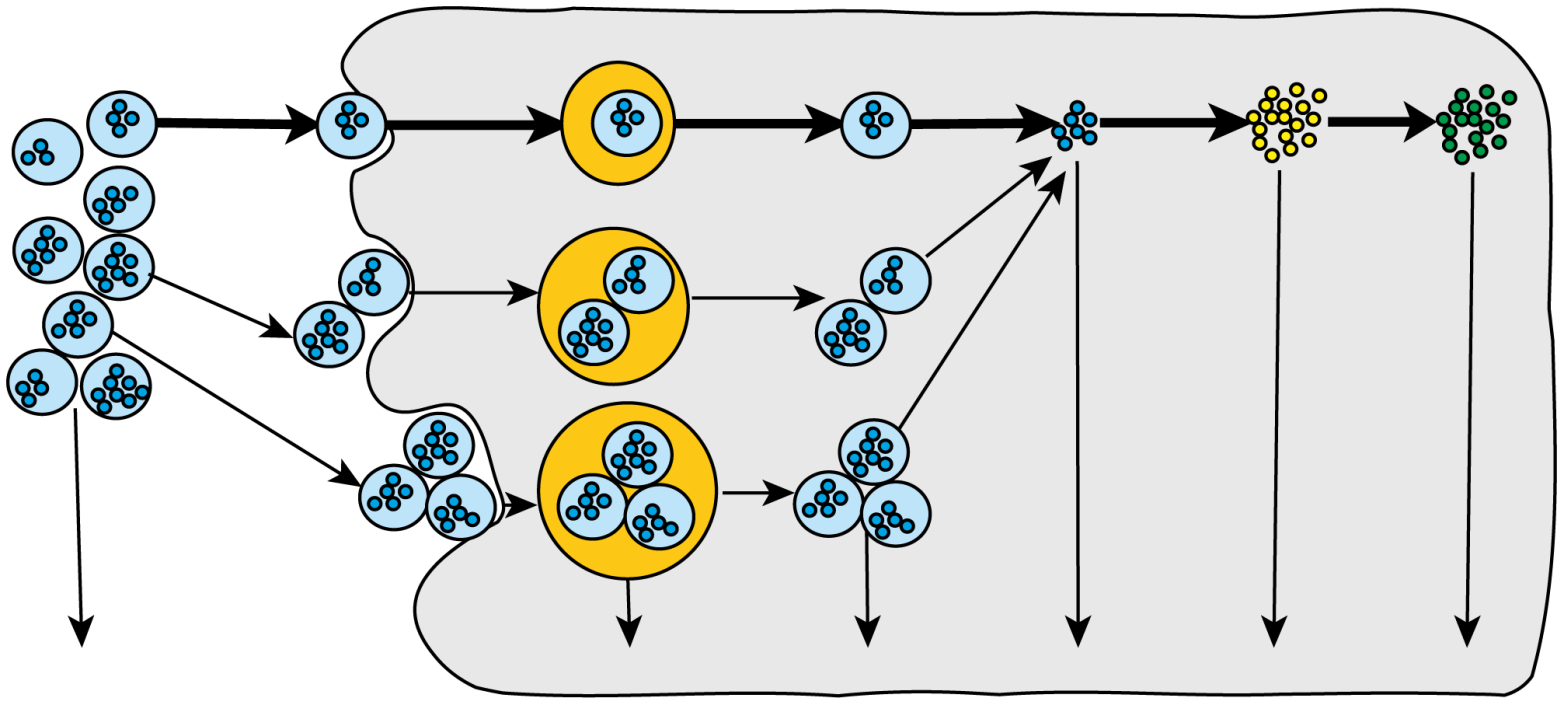
Diagram of the multiple-lipoplex transfection model. This includes the same processes as in the streamlined model, except that here the clathrin-coated pits and the endosomes can contain multiple lipoplexes.

The solution to the complexity that arises from multiple levels of structure is a key aspect of the model shown in Figure 2, so we will describe it here in very general terms. For readers who are interested in more detail, the File S1 contains the code of all versions of the model.

The initial condition of external lipoplexes provides a first example of this. In ordinary differential equations, we would use the name of the lipoplexes (Lext or L_ex_) as a variable in the equations. This variable refers to the concentration of lipoplexes, or, equivalently, the number of particles in a given volume. In an SBML-based [27] tool, this is also called a species. Now the problem here is that the lipoplexes come in different sizes, based on the number of mRNA molecules they contain. In the current experimental situation we are modeling, the lipoplexes have a mean diameter of 120 nm and a standard deviation of 10 nm. This size was determined by fluorescence correlation spectroscopy (data not shown). When we additionally take the packing density of the lipoplexes into account, this size corresponds to a mean of 350 mRNA molecules per lipoplex and lipoplex sizes ranging from 270 to 445 mRNA molecules. See Supplementary data of Leonhardt et al. [44] for a detailed description.

There are three solutions to this problem. First, we can use a tool in which we can include a parameter for the size of the lipoplex. In other words, we can write Lext(n), where n is the number of mRNA molecules, and use that in the model. Second, as an alternative, we can simply list all possible values of the size as separate species, e.g. Lext270, Lext271 … Lext445. Finally, we can apply a key simplification and assume that all lipoplexes contain exactly 350 mRNA molecules.

Next, we need to consider the endosomes. Our experience with both experimental data and modeling shows us that each endosome can only contain a small number of lipoplexes, and we are safe when we set this to an arbitrary maximum of 10. In addition, each of those lipoplexes can contain anywhere from 270 to 445 mRNA molecules. In order to list all of these cases, we would need more than 175^10^ different variables (or species), something that is clearly impossible.

The key simplification in this paper, assuming that all lipoplexes have the same size, along with listing all possible endosome sizes, makes it possible to formulate the model in SBML and use Copasi to run the simulations. We have also evaluated the use of other tools and present those results here, for the benefit of experts in those tools and modeling techniques in general. The second implementation uses Pi-Calculus-based SPiM and preserves full complexity, except that we used a smaller width for the lipoplex size distribution in order to keep the code smaller. The variable sizes of the lipoplexes are kept throughout their lifetime, and the variable sizes of pits and endosomes are represented by listing all possible values, due to limitations in formulating reactions of parameters in SPiM (as opposed to processes). The third version uses the rule-based language BioNetGen Language (BNGL) in the tool NFsim, and exposes a limitation that prevents us from using a parameter (such as the number of mRNA molecules in a lipoplex) in a reaction without setting it to an explicit value.

### Multiple-Lipoplex Model

The multiple-lipoplex model (Figure 2) follows the lines of the streamlined model (heavy arrows), but also includes the formation of clathrin-coated pits that include multiple lipoplexes.

The delivery phase is described by the following ODEs:
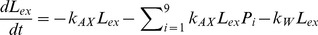
(8)

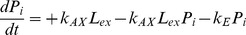
(9)

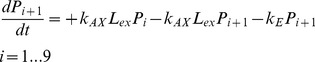
(10)

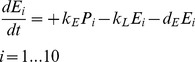
(11)

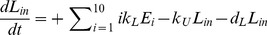
(12)and equation (5) from above, where *P_i_* is the concentration of clathrin-coated pits of size i, i.e. containing i lipoplexes, *E_i_* is the concentration of endosomes of size i, and the new rate of attachment is *k_AX_* calculated by dividing *k_A_* by the number of pits plus one, in order to assure a constant rate of attachment even when the number of pits increases. All other symbols are the same as in the streamlined model.

The expression phase is described by the same ODEs as in the streamlined model, (5), (6), and (7).

This model, in contrast to the streamlined model, includes different-sized lipoplexes, with their sizes preserved through all reactions up to unpacking. This seemingly easy extension allowing variable lipoplex sizes and variable endosome sizes leads to a severe combinatorial explosion of species and reactions. For the analysis included in this paper, we have avoided a large part of this issue by assuming that all lipoplexes have the same size. This is a very significant simplification, but nevertheless allows fairly good simulation results, and makes it possible to run simulations both deterministically and stochastically, and also to run parameter estimation.

We created 3 implementations of the model. The first is written in SBML, was run in Copasi, and assumes a very significant simplification (all liposomes have the same size); it was used for the analysis in this paper. The second is written in Pi Calculus and was run in the Stochastic Pi Machine (SPiM), and includes a limited example of variable-sized lipoplexes. The third is written in BNGL and was tested in NFSim.

### Parameter Optimization

In order to compare the model to the experimental data, the best values need to be found for the five parameters that have been left free, such as the rate of endocytosis. This requires adjusting the model to best fit the five experimental determinants, such as the dose-response relationship. However, since the experimental data is based on single-cell tracking, it includes the variance of the distributions of multiple time courses. As a result, each attempt to find a better value for the parameters requires two steps: First, it is necessary to run the simulation many times (typically 1,000–5,000) and second, to compare the distributions with the experimental data. In all cases where we compare simulation data to experimental data, we use the same analytical model for the expression phase and the same fitting procedures for both data sets, in order to make a good comparison between simulation and experiment, as reported in [44].

Since we are optimizing a stochastic model, we have chosen to use the simulated annealing algorithm. This algorithm chooses a new set of values for the parameters, based on random numbers, then runs the two steps of simulation and analysis described above, and compares the results with the experimental data. The comparison involves the current value of a “temperature” variable and the Boltzmann function in order to allow the algorithm to move away from local optima that may not be globally optimal.

The first two parameters in the model are the initial concentration of external lipoplexes and the incubation time (time until the cells are washed). These parameters are not part of the optimization process, since they are determined by experiment, but they do appear in the plots we have created of the dose-response relationship and incubation dependency, which we also compare with experimental data. In addition, we have varied these parameters as part of predictive modeling.

The parameters in the optimization process are the rates of attachment, endocytosis, and lysis, along with the rate of endosome degradation, plus the rate of GFP maturation. We optimize these five parameters to match five data points from the experimental data: The number of lipoplexes that attach to the cell surface (4–8), the dose-response curve (transfection efficiency vs. dose), the mean and variance of the onset time of GFP expression are as reported in [44], and the mean maximum GFP expression level. This gives us a good estimate of these five parameters.

The remaining parameters that need optimization are thus the rates of lysis and unpacking. Currently, we don't have a way to distinguish between delays caused by lysis vs. unpacking, so we set unpacking to be immediate. In addition, we assume no negligible degradation of lipoplexes, so we set that rate to a small number.

The values of all parameters, both fixed and fitted, are documented in Table 1. Due to the significant simplifications involved in the model, and the inherent “sloppiness” of models with this many parameters, we do not consider the parameters to be accurate measurements of the real values. The value of the model is demonstrated more by its overall performance and matching with the experimental data.

### Model Implementations

The formulation of the SBML implementation of the model is based on reactions, and is a very straightforward step from the reactions documented here. The only difference is the fact that some species are listed, such as End1…End10, instead of the subscripted notation End_i_ i = 1…10 used in the documentation.

The Pi Calculus implementation is discussed in the File S1. This implementation of the model, which was run in SPiM, deals with the variable lipoplex size by including the size as a parameter in the process. It is an implementation of the model in Pi Calculus where the number of lipoplex sizes (the width of the lipoplex size distribution) is restricted to 11, even though 175 is required. This model was run and produced the same data as the Copasi model with only 1 lipoplex size.

The BNGL implementation is discussed in the File S1. This is a prototype of an implementation of the model written in BNGL and run in NFSim. This implementation does not cover enough of the model to produce useful data.

## Results and Discussion

### Time Courses

**Figure 3:**
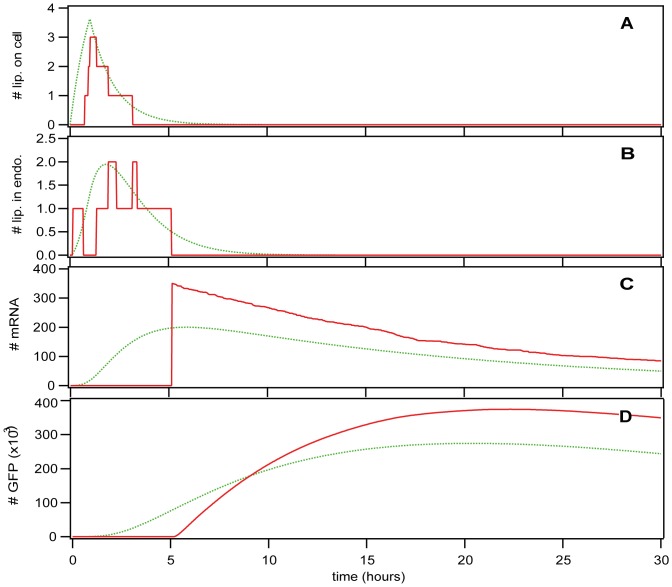
Simulation Time Courses. Green dotted (red full) line: deterministic (stochastic) simulation. A) Number of lipoplexes attached to the cell surface. B) Number of lipoplexes contained in endosomes. C) Number of mRNA molecules in the cell. D) Number of GFP molecules in the cell.

Since we are dealing with low copy numbers in the first parts of the transfection process, we need to account for the stochastic nature of them, and see how that compares with a more traditional solution to the equations. Figure 3 shows time courses created by deterministic simulation, i.e. by numerical solution of the differential equations in the green dotted line, and a typical example of time courses created by stochastic simulation, i.e. using Monte Carlo simulation via the Gillespie algorithm in the red full line. The important message in this figure is the very significant difference between deterministic and stochastic simulations. Due to the low copy numbers involved (except for GFP), the deterministic plots are not good representations of the biological reality, and they do not necessarily represent the average behavior of the stochastic simulations. However, they are sometimes useful for running early steps in the parameter estimation task. Figure 3A shows the number of lipoplexes attached to the cell surface, which grows rapidly until the cells are washed after 1 hour of incubation, and then decays exponentially as they enter the cell. Figure 3B shows the number of lipoplexes in endosomes, which demonstrates how they enter and leave the endosomes. Figure 3C shows the number of mRNA molecules, where our example of a stochastic simulation shows that 1 lipoplex (containing 350 mRNA molecules) has entered the cell; this can vary from 0 to about 5. Figure 3D shows the number of GFP molecules, which first increases after mRNA molecules appear and begin to translate, then decreases due to degradation of both mRNA and GFP.

**Figure 4:**
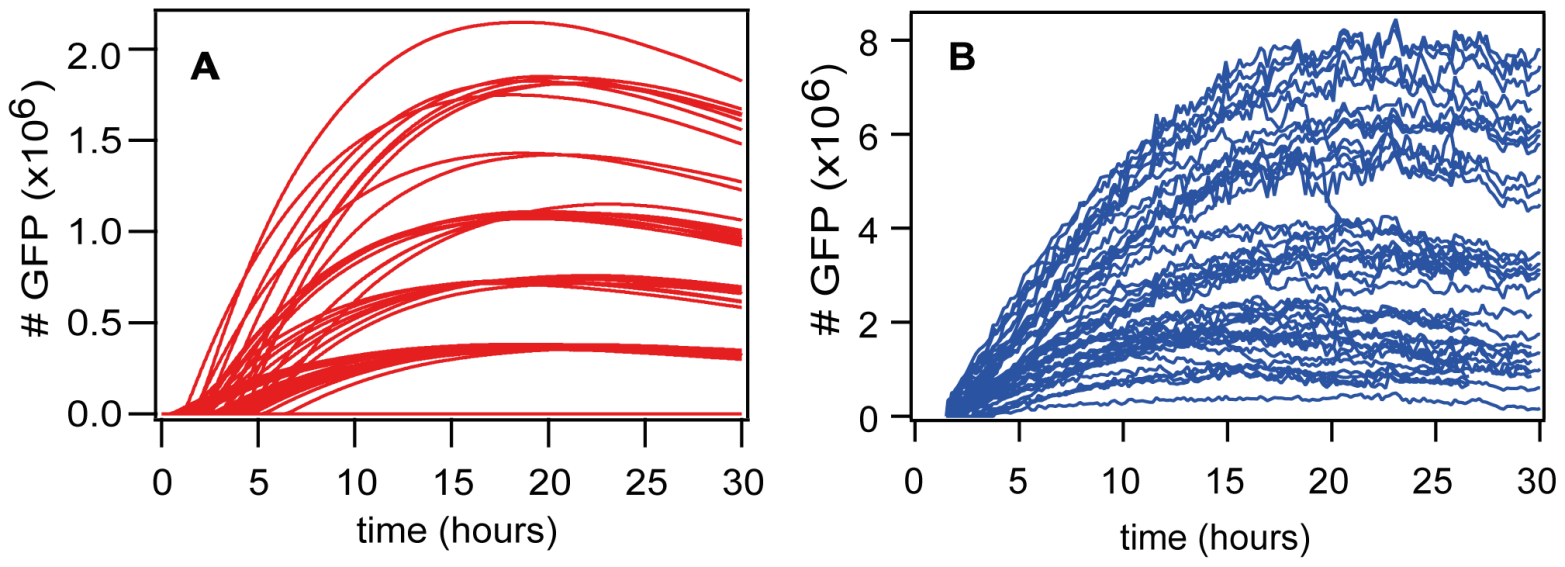
GFP expression: simulation vs. experiment. A) Computer simulation. B) Experimental time courses.

Now that we have set our focus on stochastic simulation time courses, we would like to see how they compare with the experimental data. Figure 4 is another visualization of the GFP time course presented earlier. Figure 4A shows the simulation data. The clustering of the absolute height of the curves results from the fact that mRNA molecules are delivered in “packets”, i.e. lipoplexes of size 350. We consider this to be a result of the simplification where we assumed all lipoplexes to contain exactly 350 mRNA molecules, even though the range (within one standard deviation) goes from 270 to 445. This clustering behavior was not observed in the experimental data. The horizontal axis clearly shows the variation in the onset time, and the vertical axis shows the variation in expression level (maximum GFP concentration). These two distributions will be examined in more detail below. Figure 4B shows the experimental data. In the plots, it appears as though the absolute level of GFP expression differs by a factor of 4. However, the value used for parameter optimization was the mean of the maximum GFP expression level, and that is 7.1×10^5^ in the experiment and 5.4×10^5^ in the simulation. The other values used for optimization varied much less (see Table 1). The time for reaching a peak value in Figure 4B is not easy to see, so we calculated the mean and variance of both distributions, and found that both peak at about 20 hours with a standard deviation of about 5.5 hours.

### Simulation vs. Experiment

**Figure 5:**
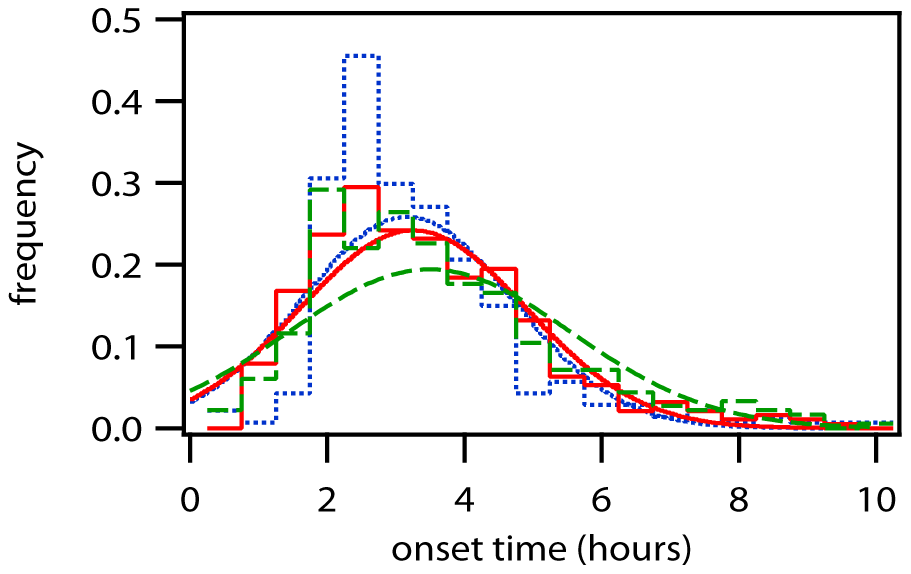
Onset time of GFP expression (Simulation vs. Experiment based on time courses shown in **Figure 4**). The curves are Gaussian curves based on mean and variance of the full distribution data (shown as a histogram). The dashed green lines show the onset times for simulation with a maturation rate (k_M_) of 9.23 taken from literature. The solid red lines show the onset times for simulation with a maturation rate (k_M_) of 5.5. The dotted blue lines show the onset times of the experimental data.

**Figure 6:**
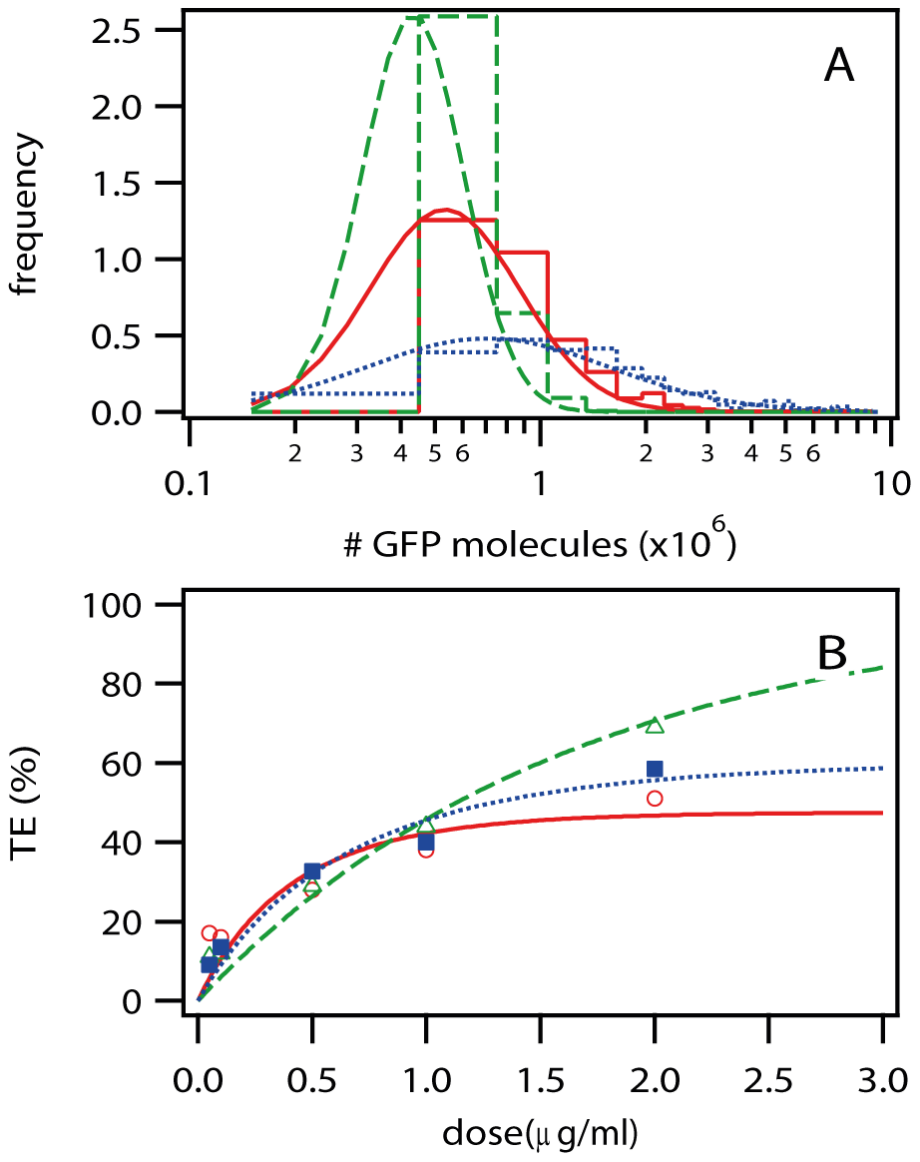
GFP expression (Simulation vs. Experiment based on time courses shown in **Figure 4**). A) Expression Level. Maximum number of GFP molecules with histograms of the distributions and lognormal fits of the histograms as curves. The dashed green lines are from a simulation of the streamlined model. The solid red lines are from a simulation of the multiple-lipoplex model. The dotted blue lines show the experimental data. B) Dose-Response Relationship. Transfection efficiency (TE) is the percentage of cells that exhibited a successful transfection, based on GFP expression. The curve was determined by varying the dosage (µg/ml) in the experiment, and the initial concentration of lipoplexes in the simulation (L_ex_). The green open triangles are from the simulation of the streamlined model, and the dashed green line is a single-Poissonian fit. The open red circles are from the simulation of the multiple-lipoplex model and the solid red line is a double-Poissonian fit. The solid blue squares are from the experimental data and the dotted blue line is a double-Poissonian fit.

In order to compare simulation with experiment, probability distributions of some of the key parameters are shown in Figures 5 and 6. In all cases, the experimental data refers to the data published in [44]. Figure 5 shows the onset time of GFP expression, which is defined as the first time where GFP can be detected, and we have measured it by fitting the analytic solution of the expression kinetics to the experimental data and the simulations using the same technique as in the original paper [44]. This makes it unnecessary to use an arbitrary threshold for GFP or to use the simple slope of the curve to determine onset time. The maturation reaction was not included in the original analysis in [44], which means that the maturation delay was included in the onset time there. The green dashed line kM = 9.23 (fitted parameters 3.5 mean and 2.1 width), from literature [48], and solid red line kM = 5.5 (fitted parameters 3.2 mean and 1.6 width), as determined by our parameter optimization. The dotted blue lines show the onset times of the experimental data (fitted with 3.1 mean and 1.5 width). The reason for the difference lies in the fact that all reactions have a small copy number, and thus a large stochastic variation, except for the maturation reaction. We know that, for Poisson processes, the mean is proportional to the number of reactants, and the width is proportional to the square root of the number of reactants, and this number is on the order of 1–100 for endocytosis, 1–100 for lysis, 1–100 for unpacking, 300–2000 for translation, and 200,000–5,000,000 for maturation. In order to match the experimental results, our optimization routine found a maturation rate of 5.5 h^−1^ or 11 min delay. In contrast, the rate of kM = 9.23 (6.5 min) from literature produces a distribution that is too wide. Maturation delays of 20 or 30 minutes also match the experimental data well. This is within the range of published EGFP maturation rates, which vary widely and go as high as a few hours due to the time required for oxidation (more details in File S1). This figure was created in the multiple-lipoplex model, but the streamlined model shows exactly the same behavior, i.e. it is capable of reproducing the experimentally-measured onset time distribution, but also needs the maturation reaction to do so.

Now that we have seen the comparison of simulation and experiment for the onset time of GFP expression, we need to look at how much GFP is created in the cells. Figure 6A shows the distribution of the maximum number of GFP molecules, as determined by fitting the analytical solution of gene expression (translation and degradation) to the data of simulation and experiment. This is the value that we use to determine the level of expression, and, along with the degradation rates, it uniquely determines the time course of GFP expression. The dashed green lines are from a simulation of the streamlined model (fitted with 4.3*10^5^ mean and 0.47 width). The solid red lines are from a simulation of the multiple-lipoplex model (fitted with 5.3*10^5^ mean and 0.69 width). The dotted blue lines show the experimental data (fitted with 7.1*10^5^ mean and 1.1 width). We can see that the simulation of the streamlined model misses the experimental results significantly, which we attribute to the fact that the streamlined model never transports more than one lipoplex per endosome. In contrast to the streamlined model, the multiple-lipoplex model allows a better match to the expression level data. The use of lognormal curves to fit the simulation and experimental data in Figure 6A is more than a convenient guide for the eye; they provide a good representation of the data, since the GFP expression is the result of multiple random processes.

Along with the maximum amount of GFP expressed, we are also interested in seeing how the amount of GFP compares with the dosage of lipoplexes, i.e. the concentration presented to the cells. Figure 6B shows the dose-response relationship, defined as transfection efficiency, i.e. percentage of cells that successfully express GFP vs. concentration of mRNA. The green open triangles are from the simulation of the streamlined model, and the dashed green line is a single-Poissonian fit (fitted parameter 1.1). The open red circles are from the simulation of the multiple-lipoplex model and the solid red line is a double-Poissonian fit (fitted parameters 1.9 and 0.6). The solid blue squares are from the experimental data and the dotted blue line is a double-Poissonian fit (fitted parameters 1.1 and 0.9). In Figure 6B, we can see that the simulation of the streamlined model is much too straight and significantly misses the shape of the experimental results, which we attribute to the fact that the streamlined model never transports more than one lipoplex per endosome. In fact, the good fit of a single Poissonian to the streamlined model is a clear indication that one of the Poissonian processes, representing the number of lipoplexes per endosome, is missing in this model. This process is referred to as L_eff_ in the original paper, and the process that is included in the streamlined model is referred to as N_eff_ [44], File S1. The dose-response relationship for the multiple-lipoplex model shows a reasonable fit to a double Poissonian and to the experimental data, and is a big improvement over the streamlined model.

We can summarize these differences by observing that the streamlined model is capable of reproducing the delay and variance of the onset time of GFP expression, but the multiple-lipoplex model is required to reproduce the dispersion of the data. In other words, multi-level modeling is necessary for reproducing the dispersion of the data, because it is the only model that includes the second Poisson process discussed in the experimental paper.

### Predictive Modeling

The power of mathematical modeling is its capability to predict the behavior of systems before running experiments. It is instructive to test the outcome of our simulation for various scenarios of practical relevance in our lab work. In the following, the red circles show the transfection efficiency (percentage of cells transfected) and the green triangles show the maximum GFP expression level.

**Figure 7:**
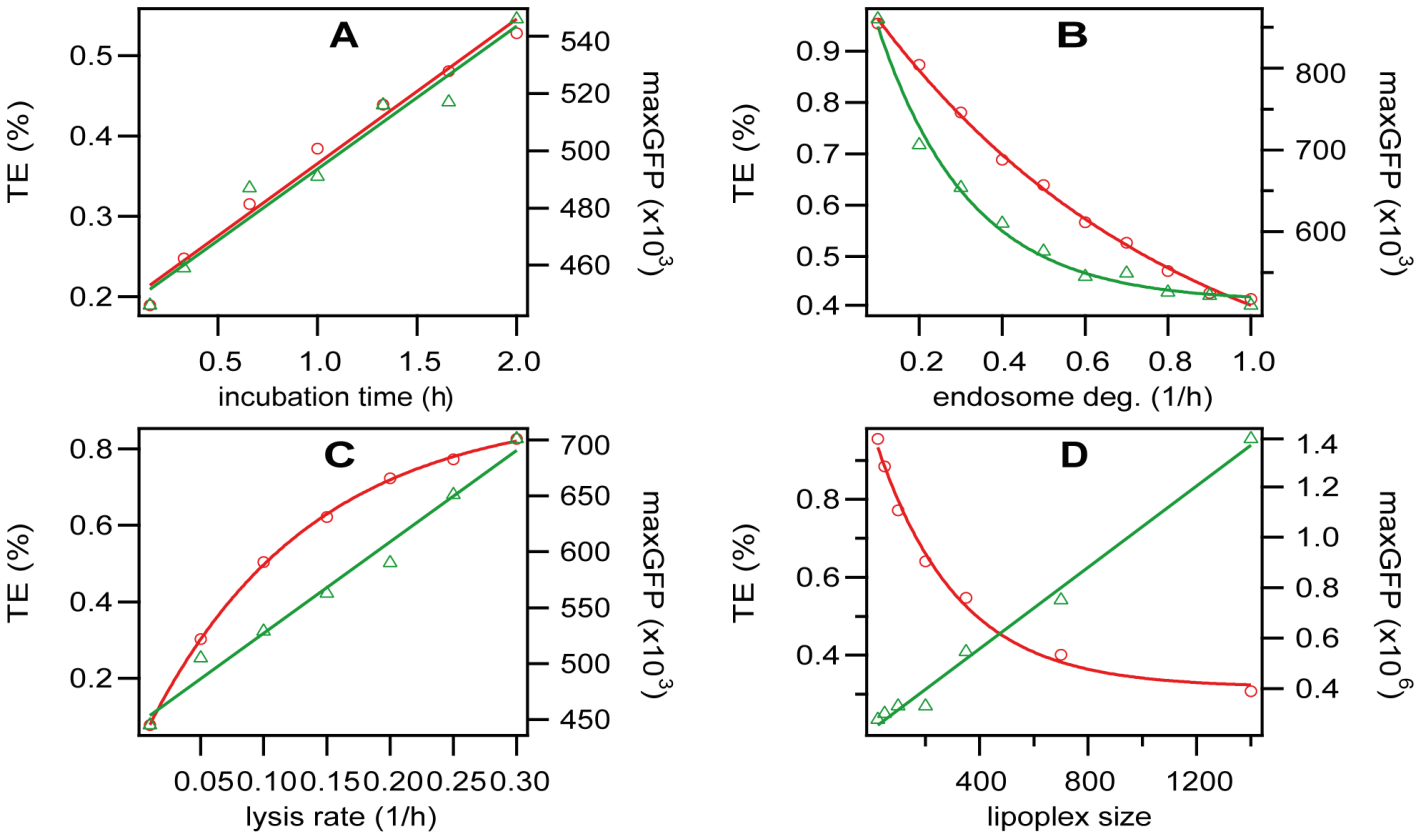
Predictive Modeling. All plots show a parameter vs. transfection efficiency (TE, red circles) and protein expression (GFP, green triangles). The lines are linear or exponential fits. A) Incubation time. B) Endosome degradation rate. C) Lysis rate. D) Lipoplex size.

For determining the dosage presented to the cells, the incubation time, i.e. the time until the cells are washed, plays an important role. Figure 7A shows the transfection efficiency (TE) and the maximal number of eGFP expressed (GFP) as a function of incubation time. The model predicts a strictly linear relation of incubation time and transfection efficiency. This outcome is due to the fact that the model assumes a constant concentration of lipoplexes in bulk and hence a constant diffusion-limited flux. Yet we expect this dependence to be only observable in a very limited time window avoiding saturation of the uptake capacity of the cells as well as the depletion of the lipoplex pool. Most importantly, however, the model does not account for increasing toxic side effects that come with increasing dose.

In this model, the endosome degradation rate is a catch-all for any kind of degradation that occurs before endosomal lysis, especially mRNA degradation, so a small endosome degradation rate should show the benefit of improved mRNA stability. Figure 7B shows the transfection efficiency (TE) and the maximal number of eGFP expressed (GFP) as a function of endosome degradation rate. The solid red and green lines are exponential fits. The exponential increase of transfection efficiency with decreasing degradation rate clearly shows the (expected) benefit of increasing the stability of mRNA. It is interesting to note that the averaged eGFP per expressing cell exhibits a steeper dependence than the fraction of transfected cells (transfection efficiency). When we extrapolate the exponential fits to the point where the endosome degradation rate is zero, we can see that the model predicts approximately 100% transfection efficiency and 1,000,000 maximum GFP for the case of perfectly stable mRNA. Extrapolation to an infinite degradation rate (absolutely unstable mRNA) predicts approximately 0% transfection efficiency as expected. However, this is only approximately 0%, and maximum GFP expression is only calculated for successfully transfected cells, so when we extrapolate to an infinite degradation rate, we see 500,000 GFP molecules per cell, but this is an artifact of the analysis. We should also recall that our model was optimized to an average of 6 lipoplexes adhering to each cell.

In order for the lipoplexes to reach the cytosol and be expressed, they first need to escape from the endosomes, which we have modeled in the endosomal lysis rate. Figure 7C shows the transfection efficiency (TE) and the maximal number of eGFP expressed (GFP) as a function of the lysis rate. The solid red line is an exponential fit while and the solid green line is a linear fit. The increase of transfection efficiency with increasing lysis rate demonstrates the (expected) improvement of transfection with increasing lysis, or endosomal escape [4], [9], [49]–[52]. We expect a similar effect when changing the attach rate via the use of magnetofection [8].

The size of the lipoplexes may have an important influence on their uptake. Figure 7D shows the transfection efficiency (TE) and the maximal number of eGFP expressed (GFP) as a function of the lipoplex size. We can see that the model predicts a higher percentage of cells transfected when the lipoplexes are smaller (but total mRNA concentration kept the same), and a higher total amount of GFP when the lipoplexes are larger. This opposing effect occurs because we assume a constant uptake rate independent of size and smaller lipoplexes mean a larger number of them, which increases the probability of successful transfection, while larger lipoplexes are capable of transporting more material. A size-independent uptake rate, however, is taken with a very big caveat. In fact, the dependence of uptake on size has been shown in experiment for gold nanoparticles [53]–[56]. Yet, there is some value to the finding that in case of variation of experiments focused on an optimal lipoplex size, in which case the size dependence might be weak, transfection efficiency and GFP expression react in the opposite direction.

## Conclusions and Outlook

We have presented a kinetic model for mRNA delivery via transfection of lipoplexes. The model consists of a chain of transfer events including lipoplex attachment, endocytosis, endosomal lysis, unpacking, translation and maturation. It was shown that parameter estimation allows direct comparison to the outcome of a single-cell transfection analysis. The model provides a kinetic model that reproduces both the delay and dispersion of the onset time and also the dose-response relationship. The delay can be reproduced using the streamlined model, but the multiple-lipoplex model, which is based on multi-level modeling, is necessary in order to reproduce the dispersion of the data. The key findings are that in order to achieve the observed level of GFP expression, as expressed in the maxGFP distribution, we need to use the multiple-lipoplex model. A multiple-lipoplex model achieves the correct width (stochastic variance) of the probability distribution for the onset time of GFP expression if the maturation reaction is included. A hallmark of the multiple-lipoplex model is its combinatorial manifold, which exceeds the capacity of ordinary modeling platforms. We showed that a reduction of the combinatorial space to a limited variance was able to approximate the shape of the dose-response relationship.

Extensions of the model that might be necessary as more refined data become available are more explicit rate equations that include cooperative behavior (Hill kinetics) or e.g. enzyme limited reactions (Michaelis Menten type kinetics). Furthermore, degradation processes could be broken down into specifically known pathways. Yet the most important uncertainty concerns the uptake process itself. The fact that we used a single, uniform rate of attachment of lipoplexes to clathrin-coated pits and that the rate of endocytosis in our model does not depend on the size of the pit is first of all due to missing quantitative data. We have assumed that endosomes first undergo lysis, then the lipoplexes are unpacked, and then the mRNA can begin translation and degradation. However, unpacking might occur within the endosome before lysis and, as mentioned earlier, mRNA degradation might begin in the endosome before lysis. Furthermore, we don't currently have a way to distinguish between a delay caused by lysis and delay caused by unpacking, so we have simplified the model to treat unpacking as an immediate reaction.

**Figure 8:**
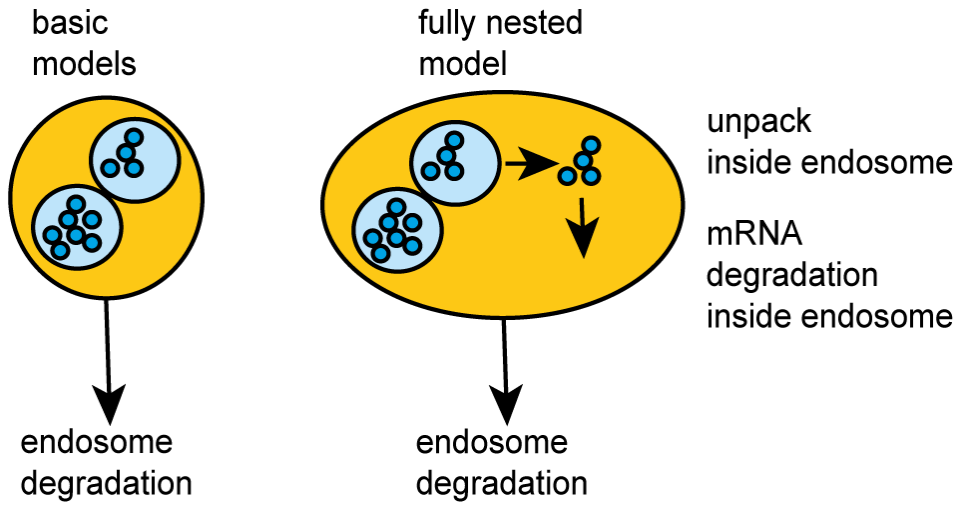
Key aspect of the fully nested transfection model. In addition to the processes in the multiple-lipoplex model, the fully nested model includes unpacking of lipoplexes and degradation of mRNA within endosomes.

A key aspect of this investigation is multi-level modeling, which leads to a combinatorial explosion of variables and reactions, but this could be solved more elegantly by a computational system that copes with it directly. However, this does not make the combinatorial explosion disappear; the burden is simply transferred from the user to the tool in the form of dynamic creation of species. The basis for this already exists in SBML, Copasi, SPiM, BioNetGen, NFsim, and ML-Rules, which introduces the concept of nested species [57], [58], meaning that one species, such as mRNA molecules, can exist and exhibit behavior within another species, such as a lipoplex or endosome. This would make it possible to formulate the model in a more elegant way, which would be easier to understand. As a second benefit, it would make it possible to remove a significant limitation of today's model, which assumes that all lipoplexes have the same size and leads to a clustering of GFP expression levels visible in Figure 4, and it would be possible to model explicit unpacking of lipoplexes and degradation of mRNA within endosomes, instead of resorting to an endosome degradation reaction, as shown in the fully nested model (Figure 8). Finally, it would also make it possible to use species as building blocks to create new ones; for example, chemical reaction networks could be used to build organelles, which could be used to build cells, etc. This type of model is often required for nanoparticle transport in general, and should provide a basis for more predictive modeling in that area.

Beside all well-founded shortcomings of the current model limitations, there is substantial value added by comparison of modeling and experimental data. The fact that data are reproduced by a set of parameters that is optimized by the same number of experimental determinants justifies our assertion that the model has significant predictive power. We have done predictive modeling by analyzing the effect of varying parameters, and the results either agree with existing experimental data (e.g. dose-response), confirm known aspects (e.g. importance of endosomal escape), or predict new effects, such as the effect that decreasing the size of the lipoplexes has on transfection efficiency and GFP expression.

With appropriate modifications, this model should be useful for new experimental work. The key parameters include the rates of attachment, endocytosis, lysis, unpacking, and the size-dependency of those rates; as new data on these parameters becomes available, this should lead to a significant improvement in the quality of the model.

## Supporting Information

File S1 **Code S1. Script for automated simulation of dose-response relationship.**
**Code S2. script for automated simulation of lipoplex size dependency.**
**Code S3. C# source code for program to set parameters in Copasi model.**
**Code S4. C# source code for program to run Copasi model multiple times and analyze results in Igor Pro.**
**Code S5. C# source code for program to run TFC.exe and optimize via simulated annealing algorithm.**
**Code S6. C# source code for program to run Copasi streamlined (reduced) model multiple times and analyze results in Igor Pro.**
**Code S7. C# source code for program to run TFRC.exe and optimize via simulated annealing algorithm.**
**Code S8. Igor Pro procedure for analyzing results of Copasi model (TFC.cps and TFC.cps).**
**Code S9. Perl script for running SPiM model.**
**Code S10. Igor Pro procedure for analyzing results of SPiM model.**
**Code S11. Igor Pro procedure for creating figures.**
**Dataset S1. Dose-response data (** **Figure 6** **).**
**Dataset S2. GFP data (** **Figure 4** **).**
**Dataset S3. Lipoplex size data (** **Figure 7** **).**
**Dataset S4. Max GFP experiment (** **Figure 5B** **).**
**Dataset S5Max GFP reduced model (** **Figure 5B** **).**
**Dataset S6. Max GFP (** **Figure 5B** **).**
**Dataset S7. Onset time experiment (** **Figure 5A** **).**
**Dataset S8. Onset time reduced model (** **Figure 5A** **).**
**Dataset S9. Onset time (** **Figure 5A** **).**
**Dataset S10. Time courses (** **Figure 3** **).**
**Model S1. Copasi model for deterministic simulation of multiple lipoplex model.**
**Model S2. SBML model for deterministic simulation of multiple lipoplex model.**
**Model S3. Copasi model for stochastic simulation of multiple lipoplex model.**
**Model S4. SBML model for stochastic simulation of multiple lipoplex model.**
**Model S5. Copasi model for deterministic simulation of streamlined (reduced) model.**
**Model S6. SBML model for deterministic simulation of streamlined (reduced) model.**
**Model S7. Copasi model for stochastic simulation of streamlined (reduced) model.**
**Model S8. SBML model for stochastic simulation of streamlined (reduced) model.**
**Model S9. SPiM model.**
**Model S10. Version 1 of BNGL (BioNetGenML) model for NFSim.**
**Model S11. SBML model for BNGL.**
**Text S1. Detailed model description.**
**Text S2. ODEs as created by Copasi in TeX format.**
**Text S3. ODEs imbedded in LaTeX document file.**
**Text S4. ODEs in PDF format (from LaTeX).**
(ZIP)

## References

[1] Neumann B, Held M, Liebel U, Erfle H, Rogers P, et al (2006) High-throughput RNAi screening by time-lapse imaging of live human cells. Nat Meth 3: 385–390. 10.1038/nmeth876 16628209

[2] Rantala JK, Mäkelä R, Aaltola A-R, Laasola P, Mpindi J-P, et al (2011) A cell spot microarray method for production of high density siRNA transfection microarrays. BMC genomics 12: 162. 21443765 10.1186/1471-2164-12-162 PMC3073923

[3] Rinaudo K, Bleris L, Maddamsetti R, Subramanian S, Weiss R, et al (2007) A universal RNAi-based logic evaluator that operates in mammalian cells. Nat Biotech 25: 795–801. 10.1038/nbt1307 17515909

[4] Tseng Y-C, Mozumdar S, Huang L (2009) Lipid-based systemic delivery of siRNA. Advanced drug delivery reviews 61: 721–731. 19328215 10.1016/j.addr.2009.03.003 PMC3172140

[5] Debus H, Baumhof P, Probst J, Kissel T (2010) Delivery of messenger RNA using poly(ethylene imine)–poly(ethylene glycol)-copolymer blends for polyplex formation: Biophysical characterization and in vitro transfection properties. Journal of Controlled Release 148: 334–343. 20854856 10.1016/j.jconrel.2010.09.007

[6] Kamiya H, Akita H, Harashima H (2003) Pharmacokinetic and pharmacodynamic considerations in gene therapy. Drug Discovery Today 8: 990–996. 14643162 10.1016/s1359-6446(03)02889-7

[7] Malone RW, Felgner PL, Verma IM (1989) Cationic liposome-mediated RNA transfection. Proceedings of the National Academy of Sciences 86: 6077–6081. 10.1073/pnas.86.16.6077 PMC297778 2762315

[8] Sauer AM, de Bruin KG, Ruthardt N, Mykhaylyk O, Plank C, et al (2009) Dynamics of magnetic lipoplexes studied by single particle tracking in living cells. Journal of Controlled Release 137: 136–145. 19358868 10.1016/j.jconrel.2009.04.003

[9] Schloßbauer A, Sauer AM, Cauda V, Schmidt A, Engelke H, et al (2012) Cascaded Photoinduced Drug Delivery to Cells from Multifunctional Core–Shell Mesoporous Silica. Advanced Healthcare Materials 1: 316–320. 23184746 10.1002/adhm.201100033

[10] Tachibana R, Harashima H, Shinohara Y, Kiwada H (2001) Quantitative studies on the nuclear transport of plasmid DNA and gene expression employing nonviral vectors. Advanced drug delivery reviews 52: 219–226. 11718946 10.1016/s0169-409x(01)00211-3

[11] Mac Gabhann F, Annex BH, Popel AS (2010) Gene therapy from the perspective of systems biology. Current opinion in molecular therapeutics 12: 570. 20886389 PMC3021921

[12] Morille M, Passirani C, Vonarbourg A, Clavreul A, Benoit J-P (2008) Progress in developing cationic vectors for non-viral systemic gene therapy against cancer. Biomaterials 29: 3477–3496. 18499247 10.1016/j.biomaterials.2008.04.036

[13] Nguyen J, Szoka FC (2012) Nucleic Acid Delivery: The Missing Pieces of the Puzzle? Accounts of Chemical Research 45: 1153–1162. 22428908 10.1021/ar3000162 PMC3399092

[14] Pedroso de Lima MC, Simões S, Pires P, Faneca H, Düzgüneş N (2001) Cationic lipid–DNA complexes in gene delivery: from biophysics to biological applications. Advanced drug delivery reviews 47: 277–294. 11311996 10.1016/s0169-409x(01)00110-7

[15] Rädler JO, Koltover I, Salditt T, Safinya CR (1997) Structure of DNA-Cationic Liposome Complexes: DNA Intercalation in Multilamellar Membranes in Distinct Interhelical Packing Regimes. Science 275: 810–814. 9012343 10.1126/science.275.5301.810

[16] Safinya CR (2001) Structures of lipid–DNA complexes: supramolecular assembly and gene delivery. Current Opinion in Structural Biology 11: 440–448. 11495736 10.1016/s0959-440x(00)00230-x

[17] Schaffert D, Wagner E (2008) Gene therapy progress and prospects: synthetic polymer-based systems. Gene Ther 15: 1131–1138. 18528432 10.1038/gt.2008.105

[18] Shahrezaei V, Swain PS (2008) Analytical distributions for stochastic gene expression. Proceedings of the National Academy of Sciences 105: 17256–17261. 10.1073/pnas.0803850105 PMC2582303 18988743

[19] Varga CM, Hong K, Lauffenburger DA (2001) Quantitative Analysis of Synthetic Gene Delivery Vector Design Properties. Mol Ther 4: 438–446. 11708880 10.1006/mthe.2001.0475

[20] Varga CM, Tedford NC, Thomas M, Klibanov AM, Griffith LG, et al (2005) Quantitative comparison of polyethylenimine formulations and adenoviral vectors in terms of intracellular gene delivery processes. Gene Ther 12: 1023–1032. 15815703 10.1038/sj.gt.3302495

[21] Dinh AT, Pangarkar C, Theofanous T, Mitragotri S (2007) Understanding intracellular transport processes pertinent to synthetic gene delivery via stochastic simulations and sensitivity analyses. Biophys J 92: 831–846. 17085500 10.1529/biophysj.106.095521 PMC1779970

[22] Summers HD, Rees P, Holton MD, Rowan Brown M, Chappell SC, et al (2011) Statistical analysis of nanoparticle dosing in a dynamic cellular system. Nat Nano 6: 170–174. 10.1038/nnano.2010.277 21258333

[23] Schwake G, Youssef S, Kuhr JT, Gude S, David MP, et al (2010) Predictive modeling of non-viral gene transfer. Biotechnol Bioeng 105: 805–813. 19953668 10.1002/bit.22604

[24] Locke JC, Elowitz MB (2009) Using movies to analyse gene circuit dynamics in single cells. Nat Rev Microbiol 7: 383–392. 19369953 10.1038/nrmicro2056 PMC2853934

[25] Fisher J, Henzinger TA (2007) Executable cell biology. Nat Biotechnol 25: 1239–1249. 17989686 10.1038/nbt1356

[26] Mahmutovic A, Fange D, Berg OG, Elf J (2012) Lost in presumption: stochastic reactions in spatial models. Nature Methods 9: 1163–1166. 23223170 10.1038/nmeth.2253

[27] Hucka M, Bergmann F, Hoops S, Keating S, Sahle S, et al. (2010) The Systems Biology Markup Language (SBML): Language Specification for Level 3 Version 1 Core. Nature Precedings. 10.2390/biecoll-jib-2015-266 PMC5451324 26528564

[28] Funahashi A, Morohashi M, Kitano H, Tanimura N (2003) CellDesigner: a process diagram editor for gene-regulatory and biochemical networks. Biosilico 1: 159–162.

[29] Hoops S, Sahle S, Gauges R, Lee C, Pahle J, et al (2006) COPASI–a COmplex PAthway SImulator. Bioinformatics 22: 3067–3074. 17032683 10.1093/bioinformatics/btl485

[30] Milner R (1999) Communicating and Mobile Systems: The Pi Calculus: Cambridge University Press.

[31] Priami C (1995) Stochastic π-calculus. The Computer Journal 38: 578–589.

[32] Priami C, Regev A, Shapiro E, Silverman W (2001) Application of a stochastic name-passing calculus to representation and simulation of molecular processes. Information processing letters 80: 25–31.

[33] Regev A, Shapiro E (2002) Cellular abstractions: Cells as computation. Nature 419: 343–343. 12353013 10.1038/419343a

[34] Regev A, Shapiro E (2004) The π-calculus as an abstraction for biomolecular systems. Modelling in Molecular Biology: 219–266.

[35] Regev A, Silverman W, Shapiro E (2001) Representation and simulation of biochemical processes using the-calculus process algebra. pp. 459–470. 10.1142/9789814447362_0045 11262964

[36] Regev A, Panina EM, Silverman W, Cardelli L, Shapiro E (2004) BioAmbients: an abstraction for biological compartments. Theoretical Computer Science 325: 141–167.

[37] Guerriero M, Priami C, Romanel A (2007) Modeling static biological compartments with beta-binders. Algebraic Biology: 247–261.

[38] Guerriero ML, Priami C, Romanel A (2006) Beta-binders with static compartments.

[39] Priami C, Quaglia P (2005) Beta binders for biological interactions. Springer.pp. 20–33.

[40] Phillips A, Cardelli L. Efficient, correct simulation of biological processes in the stochastic pi-calculus; 2007. Springer.pp. 184–199.

[41] Faeder JR, Blinov ML, Hlavacek WS (2009) Rule-based modeling of biochemical systems with BioNetGen. Systems biology: Springer.pp. 113–167. 10.1007/978-1-59745-525-1_5 19399430

[42] Sneddon MW, Faeder JR, Emonet T (2011) Efficient modeling, simulation and coarse-graining of biological complexity with NFsim. Nat Meth 8: 177–183. 10.1038/nmeth.1546 21186362

[43] Dobay MPD, Alberola AP, Mendoza ER, Rädler JO (2012) Modeling nanoparticle uptake and intracellular distribution using stochastic process algebras. Journal of Nanoparticle Research 14.

[44] Leonhardt C, Schwake G, Stögbauer TR, Rappl S, Kuhr J-T, et al. (2013) Single-cell mRNA transfection studies: delivery, kinetics and statistics by numbers. Nanomedicine: Nanotechnology, Biology and Medicine. 10.1016/j.nano.2013.11.008 24333584

[45] Andries O, De Filette M, Rejman J, De Smedt SC, Demeester J, et al (2012) Comparison of the Gene Transfer Efficiency of mRNA/GL67 and pDNA/GL67 Complexes in Respiratory Cells. Molecular Pharmaceutics 9: 2136–2145. 22676473 10.1021/mp200604h

[46] Tavernier G, Andries O, Demeester J, Sanders NN, De Smedt SC, et al (2011) mRNA as gene therapeutic: How to control protein expression. Journal of Controlled Release 150: 238–247. 20970469 10.1016/j.jconrel.2010.10.020

[47] Khalil IA, Kogure K, Akita H, Harashima H (2006) Uptake pathways and subsequent intracellular trafficking in nonviral gene delivery. Pharmacological reviews 58: 32–45. 16507881 10.1124/pr.58.1.8

[48] Megerle JA, Fritz G, Gerland U, Jung K, Rädler JO (2008) Timing and Dynamics of Single Cell Gene Expression in the Arabinose Utilization System. Biophys J 95: 2103–2115. 18469087 10.1529/biophysj.107.127191 PMC2483754

[49] Chan C-L, Majzoub RN, Shirazi RS, Ewert KK, Chen Y-J, et al (2012) Endosomal escape and transfection efficiency of PEGylated cationic liposome–DNA complexes prepared with an acid-labile PEG-lipid. Biomaterials 33: 4928–4935. 22469293 10.1016/j.biomaterials.2012.03.038 PMC3337860

[50] Dominska M, Dykxhoorn DM (2010) Breaking down the barriers: siRNA delivery and endosome escape. Journal of cell science 123: 1183–1189. 20356929 10.1242/jcs.066399

[51] Sauer AM, Schlossbauer A, Ruthardt N, Cauda V, Bein T, et al (2010) Role of endosomal escape for disulfide-based drug delivery from colloidal mesoporous silica evaluated by live-cell imaging. Nano Lett 10: 3684–3691. 20677799 10.1021/nl102180s

[52] Xu Y, Szoka FC (1996) Mechanism of DNA Release from Cationic Liposome/DNA Complexes Used in Cell Transfection†,‡. Biochemistry 35: 5616–5623. 8639519 10.1021/bi9602019

[53] Chithrani BD, Chan WCW (2007) Elucidating the Mechanism of Cellular Uptake and Removal of Protein-Coated Gold Nanoparticles of Different Sizes and Shapes. Nano Lett 7: 1542–1550. 17465586 10.1021/nl070363y

[54] Chithrani BD, Ghazani AA, Chan WCW (2006) Determining the Size and Shape Dependence of Gold Nanoparticle Uptake into Mammalian Cells. Nano Lett 6: 662–668. 16608261 10.1021/nl052396o

[55] Gao H, Shi W, Freund LB (2005) Mechanics of receptor-mediated endocytosis. Proceedings of the National Academy of Sciences of the United States of America 102: 9469–9474. 15972807 10.1073/pnas.0503879102 PMC1172266

[56] Jiang W, KimBetty YS, Rutka JT, ChanWarren CW (2008) Nanoparticle-mediated cellular response is size-dependent. Nat Nano 3: 145–150. 10.1038/nnano.2008.30 18654486

[57] Faeder JR (2011) Toward a comprehensive language for biological systems. BMC biology 9: 68. 22005092 10.1186/1741-7007-9-68 PMC3195790

[58] Maus C, Rybacki S, Uhrmacher AM (2011) Rule-based multi-level modeling of cell biological systems. BMC Syst Biol 5: 166. 22005019 10.1186/1752-0509-5-166 PMC3306009

[59] Zhou J, Yockman J, Kim S, Kern S (2007) Intracellular Kinetics of Non-Viral Gene Delivery Using Polyethylenimine Carriers. Pharmaceutical Research 24: 1079–1087. 17387605 10.1007/s11095-006-9229-5

[60] Kremers G-J, Goedhart J, van den Heuvel DJ, Gerritsen HC, Gadella TWJ (2007) Improved Green and Blue Fluorescent Proteins for Expression in Bacteria and Mammalian Cells. Biochemistry 46: 3775–3783. 17323929 10.1021/bi0622874

[61] Reid BG, Flynn GC (1997) Chromophore Formation in Green Fluorescent Protein. Biochemistry 36: 6786–6791. 9184161 10.1021/bi970281w

[62] Sniegowski JA, Lappe JW, Patel HN, Huffman HA, Wachter RM (2005) Base catalysis of chromophore formation in Arg96 and Glu222 variants of green fluorescent protein. Journal of Biological Chemistry 280: 26248–26255. 15888441 10.1074/jbc.M412327200

[63] Tsien RY (1998) The green fluorescent protein. Annu Rev Biochem 67: 509–544. 9759496 10.1146/annurev.biochem.67.1.509

