# Supplementary material for: Multi-Level Kinetic Model of mRNA Delivery via Transfection of Lipoplexes
Source: PLoS One. 2014 Sep 19;9(9):e107148. doi: 10.1371/journal.pone.0107148 (PMC4169511; doi:10.1371/journal.pone.0107148)
Supplement: File S1 — Code S1. Script for automated simulation of dose-response relationship. Code S2. script for automated simulation of lipoplex size dependency. Code S3. C# source code for program to set parameters in Copasi model. Code S4. C# source code for program to run Copasi model multiple times and analyze results in Igor Pro. Code S5. C# source code for program to run TFC.exe and optimize via simulated annealing algorithm. Code S6. C# source code for program to run Copasi streamlined (reduced) model multiple times and analyze results in Igor Pro. Code S7. C# source code for program to run TFRC.exe and optimize via simulated annealing algorithm. Code S8. Igor Pro procedure for analyzing results of Copasi model (TFC.cps and TFC.cps). Code S9. Perl script for running SPiM model. Code S10. Igor Pro procedure for analyzing results of SPiM model. Code S11. Igor Pro procedure for creating figures. Dataset S1. Dose-response data ( Figure 6 ). Dataset S2. GFP data ( Figure 4 ). Dataset S3. Lipoplex size data ( Figure 7 ). Dataset S4. Max GFP experiment ( Figure 5B ). Dataset S5Max GFP reduced model ( Figure 5B ). Dataset S6. Max GFP ( Figure 5B ). Dataset S7. Onset time experiment ( Figure 5A ). Dataset S8. Onset time reduced model ( Figure 5A ). Dataset S9. Onset time ( Figure 5A ). Dataset S10. Time courses ( Figure 3 ). Model S1. Copasi model for deterministic simulation of multiple lipoplex model. Model S2. SBML model for deterministic simulation of multiple lipoplex model. Model S3. Copasi model for stochastic simulation of multiple lipoplex model. Model S4. SBML model for stochastic simulation of multiple lipoplex model. Model S5. Copasi model for deterministic simulation of streamlined (reduced) model. Model S6. SBML model for deterministic simulation of streamlined (reduced) model. Model S7. Copasi model for stochastic simulation of streamlined (reduced) model. Model S8. SBML model for stochastic simulation of streamlined (reduced) model. Model S9. SPiM model. Model S10. Versio [file pone.0107148.s001.zip › TextS004.pdf]

$$
\begin{aligned}
\frac{\mathrm{d}([\mathrm{Lext}]\cdot V_c)}{\mathrm{d}t} &= -V_c\cdot(\mathrm{kAX}\cdot[\mathrm{Lext}]) \\
&-V_c\cdot(\mathrm{kAX}\cdot[\mathrm{Lext}]\cdot[\mathrm{Pit1}]) \\
&-V_c\cdot(\mathrm{kAX}\cdot[\mathrm{Lext}]\cdot[\mathrm{Pit2}]) \\
&-V_c\cdot(\mathrm{kAX}\cdot[\mathrm{Lext}]\cdot[\mathrm{Pit3}]) \\
&-V_c\cdot(\mathrm{kAX}\cdot[\mathrm{Lext}]\cdot[\mathrm{Pit4}]) \\
&-V_c\cdot(\mathrm{kAX}\cdot[\mathrm{Lext}]\cdot[\mathrm{Pit5}]) \\
&-V_c\cdot(\mathrm{kAX}\cdot[\mathrm{Lext}]\cdot[\mathrm{Pit6}]) \\
&-V_c\cdot(\mathrm{kAX}\cdot[\mathrm{Lext}]\cdot[\mathrm{Pit7}]) \\
&-V_c\cdot(\mathrm{kAX}\cdot[\mathrm{Lext}]\cdot[\mathrm{Pit8}]) \\
&-V_c\cdot(\mathrm{kAX}\cdot[\mathrm{Lext}]\cdot[\mathrm{Pit9}]) \\
&-V_c\cdot(\mathrm{kW}\cdot[\mathrm{Lext}])
\end{aligned}
$$

$$
\begin{aligned}
\frac{\mathrm{d}([\mathrm{Pit1}]\cdot V_c)}{\mathrm{d}t} &= +V_c\cdot(\mathrm{kAX}\cdot[\mathrm{Lext}]) \\
&-V_c\cdot(\mathrm{kAX}\cdot[\mathrm{Lext}]\cdot[\mathrm{Pit1}]) \\
&-V_c\cdot(\mathrm{kE}\cdot[\mathrm{Pit1}])
\end{aligned}
$$

$$
\begin{aligned}
\frac{\mathrm{d}([\mathrm{Pit2}]\cdot V_c)}{\mathrm{d}t} &= +V_c\cdot(\mathrm{kAX}\cdot[\mathrm{Lext}]\cdot[\mathrm{Pit1}]) \\
&-V_c\cdot(\mathrm{kE}\cdot[\mathrm{Pit2}]) \\
&-V_c\cdot(\mathrm{kAX}\cdot[\mathrm{Lext}]\cdot[\mathrm{Pit2}])
\end{aligned}
$$

$$
\begin{aligned}
\frac{\mathrm{d}([\mathrm{Pit3}]\cdot V_c)}{\mathrm{d}t} &= -V_c\cdot(\mathrm{kE}\cdot[\mathrm{Pit3}]) \\
&+V_c\cdot(\mathrm{kAX}\cdot[\mathrm{Lext}]\cdot[\mathrm{Pit2}]) \\
&-V_c\cdot(\mathrm{kAX}\cdot[\mathrm{Lext}]\cdot[\mathrm{Pit3}])
\end{aligned}
$$

$$
\begin{aligned}
\frac{\mathrm{d}([\mathrm{Pit4}]\cdot V_c)}{\mathrm{d}t} &= -V_c\cdot(\mathrm{kE}\cdot[\mathrm{Pit4}]) \\
&+V_c\cdot(\mathrm{kAX}\cdot[\mathrm{Lext}]\cdot[\mathrm{Pit3}]) \\
&-V_c\cdot(\mathrm{kAX}\cdot[\mathrm{Lext}]\cdot[\mathrm{Pit4}])
\end{aligned}
$$

$$
\begin{aligned}
\frac{\mathrm{d}([\mathrm{Pit5}]\cdot V_c)}{\mathrm{d}t} &= -V_c\cdot(\mathrm{kE}\cdot[\mathrm{Pit5}]) \\
&+V_c\cdot(\mathrm{kAX}\cdot[\mathrm{Lext}]\cdot[\mathrm{Pit4}]) \\
&-V_c\cdot(\mathrm{kAX}\cdot[\mathrm{Lext}]\cdot[\mathrm{Pit5}])
\end{aligned}
$$

$$
\begin{aligned}
\frac{\mathrm{d}([\mathrm{Pit6}]\cdot V_c)}{\mathrm{d}t} &= -V_c\cdot(\mathrm{kE}\cdot[\mathrm{Pit6}]) \\
&+V_c\cdot(\mathrm{kAX}\cdot[\mathrm{Lext}]\cdot[\mathrm{Pit5}]) \\
&-V_c\cdot(\mathrm{kAX}\cdot[\mathrm{Lext}]\cdot[\mathrm{Pit6}])
\end{aligned}
$$

$$\frac{\mathrm{d}([\text{Pit7}] \cdot V_c)}{\mathrm{d}t} = -V_c \cdot (\text{kE} \cdot [\text{Pit7}]) + V_c \cdot (\text{kAX} \cdot [\text{Lext}] \cdot [\text{Pit6}]) - V_c \cdot (\text{kAX} \cdot [\text{Lext}] \cdot [\text{Pit7}])$$

$$\frac{\mathrm{d}([\text{Pit8}] \cdot V_c)}{\mathrm{d}t} = -V_c \cdot (\text{kE} \cdot [\text{Pit8}]) + V_c \cdot (\text{kAX} \cdot [\text{Lext}] \cdot [\text{Pit7}]) - V_c \cdot (\text{kAX} \cdot [\text{Lext}] \cdot [\text{Pit8}])$$

$$\frac{\mathrm{d}([\text{Pit9}] \cdot V_c)}{\mathrm{d}t} = -V_c \cdot (\text{kE} \cdot [\text{Pit9}]) + V_c \cdot (\text{kAX} \cdot [\text{Lext}] \cdot [\text{Pit8}]) - V_c \cdot (\text{kAX} \cdot [\text{Lext}] \cdot [\text{Pit9}])$$

$$\frac{\mathrm{d}([\text{Pit10}] \cdot V_c)}{\mathrm{d}t} = -V_c \cdot (\text{kE} \cdot [\text{Pit10}]) + V_c \cdot (\text{kAX} \cdot [\text{Lext}] \cdot [\text{Pit9}])$$

$$\frac{\mathrm{d}([\text{End2}] \cdot V_c)}{\mathrm{d}t} = +V_c \cdot (\text{kE} \cdot [\text{Pit2}]) - V_c \cdot (\text{kL} \cdot [\text{End2}]) - V_c \cdot (\text{dE} \cdot [\text{End2}])$$

$$\frac{\mathrm{d}([\text{End1}] \cdot V_c)}{\mathrm{d}t} = +V_c \cdot (\text{kE} \cdot [\text{Pit1}]) - V_c \cdot (\text{kL} \cdot [\text{End1}]) - V_c \cdot (\text{dE} \cdot [\text{End1}])$$

$$\frac{\mathrm{d}([\text{End3}] \cdot V_c)}{\mathrm{d}t} = +V_c \cdot (\text{kE} \cdot [\text{Pit3}]) - V_c \cdot (\text{kL} \cdot [\text{End3}]) - V_c \cdot (\text{dE} \cdot [\text{End3}])$$

$$\frac{\mathrm{d}([\text{End4}] \cdot V_c)}{\mathrm{d}t} = +V_c \cdot (\text{kE} \cdot [\text{Pit4}]) - V_c \cdot (\text{kL} \cdot [\text{End4}]) - V_c \cdot (\text{dE} \cdot [\text{End4}])$$

$$\frac{\mathrm{d}([\text{End5}] \cdot V_c)}{\mathrm{d}t} = +V_c \cdot (\text{kE} \cdot [\text{Pit5}]) - V_c \cdot (\text{kL} \cdot [\text{End5}]) - V_c \cdot (\text{dE} \cdot [\text{End5}])$$

$$\frac{\mathrm{d}([\text{End6}] \cdot V_c)}{\mathrm{d}t} = +V_c \cdot (\text{kE} \cdot [\text{Pit6}]) - V_c \cdot (\text{kL} \cdot [\text{End6}]) - V_c \cdot (\text{dE} \cdot [\text{End6}])$$

$$\frac{\mathrm{d}([\mathrm{End7}] \cdot V_c)}{\mathrm{d}t} = + V_c \cdot (\mathrm{kE} \cdot [\mathrm{Pit7}]) - V_c \cdot (\mathrm{kL} \cdot [\mathrm{End7}]) - V_c \cdot (\mathrm{dE} \cdot [\mathrm{End7}])$$

$$\frac{\mathrm{d}([\mathrm{End8}] \cdot V_c)}{\mathrm{d}t} = + V_c \cdot (\mathrm{kE} \cdot [\mathrm{Pit8}]) - V_c \cdot (\mathrm{kL} \cdot [\mathrm{End8}]) - V_c \cdot (\mathrm{dE} \cdot [\mathrm{End8}])$$

$$\frac{\mathrm{d}([\mathrm{End9}] \cdot V_c)}{\mathrm{d}t} = + V_c \cdot (\mathrm{kE} \cdot [\mathrm{Pit9}]) - V_c \cdot (\mathrm{kL} \cdot [\mathrm{End9}]) - V_c \cdot (\mathrm{dE} \cdot [\mathrm{End9}])$$

$$\frac{\mathrm{d}([\mathrm{End10}] \cdot V_c)}{\mathrm{d}t} = + V_c \cdot (\mathrm{kE} \cdot [\mathrm{Pit10}]) - V_c \cdot (\mathrm{dE} \cdot [\mathrm{End10}]) - V_c \cdot (\mathrm{kL} \cdot [\mathrm{End10}])$$

$$\frac{\mathrm{d}([\mathrm{Lint}] \cdot V_c)}{\mathrm{d}t} = + V_c \cdot (\mathrm{kL} \cdot [\mathrm{End1}]) + 2 \cdot V_c \cdot (\mathrm{kL} \cdot [\mathrm{End2}]) + 3 \cdot V_c \cdot (\mathrm{kL} \cdot [\mathrm{End3}]) + 4 \cdot V_c \cdot (\mathrm{kL} \cdot [\mathrm{End4}]) + 5 \cdot V_c \cdot (\mathrm{kL} \cdot [\mathrm{End5}]) + 6 \cdot V_c \cdot (\mathrm{kL} \cdot [\mathrm{End6}]) + 7 \cdot V_c \cdot (\mathrm{kL} \cdot [\mathrm{End7}]) + 8 \cdot V_c \cdot (\mathrm{kL} \cdot [\mathrm{End8}]) + 9 \cdot V_c \cdot (\mathrm{kL} \cdot [\mathrm{End9}]) - V_c \cdot (\mathrm{kU} \cdot [\mathrm{Lint}]) - V_c \cdot (\mathrm{dL} \cdot [\mathrm{Lint}]) + 10 \cdot V_c \cdot (\mathrm{kL} \cdot [\mathrm{End10}])$$

$$\frac{\mathrm{d}([\mathrm{mRNA}] \cdot V_c)}{\mathrm{d}t} = + 350 \cdot V_c \cdot (\mathrm{kU} \cdot [\mathrm{Lint}]) - V_c \cdot (\mathrm{dM} \cdot [\mathrm{mRNA}])$$

$$\frac{\mathrm{d}([\mathrm{GFP}] \cdot V_c)}{\mathrm{d}t} = + V_c \cdot (\mathrm{kTL} \cdot [\mathrm{mRNA}]) - V_c \cdot (\mathrm{dG} \cdot [\mathrm{GFP}])$$

$$\frac{\mathrm{d}([\mathrm{LC}] \cdot V_c)}{\mathrm{d}t} = +V_c \cdot (\mathrm{kAX} \cdot [\mathrm{Lext}])$$
$$+V_c \cdot (\mathrm{kAX} \cdot [\mathrm{Lext}] \cdot [\mathrm{Pit1}])$$
$$+V_c \cdot (\mathrm{kAX} \cdot [\mathrm{Lext}] \cdot [\mathrm{Pit2}])$$
$$+V_c \cdot (\mathrm{kAX} \cdot [\mathrm{Lext}] \cdot [\mathrm{Pit3}])$$
$$+V_c \cdot (\mathrm{kAX} \cdot [\mathrm{Lext}] \cdot [\mathrm{Pit4}])$$
$$+V_c \cdot (\mathrm{kAX} \cdot [\mathrm{Lext}] \cdot [\mathrm{Pit5}])$$
$$+V_c \cdot (\mathrm{kAX} \cdot [\mathrm{Lext}] \cdot [\mathrm{Pit6}])$$
$$+V_c \cdot (\mathrm{kAX} \cdot [\mathrm{Lext}] \cdot [\mathrm{Pit7}])$$
$$+V_c \cdot (\mathrm{kAX} \cdot [\mathrm{Lext}] \cdot [\mathrm{Pit8}])$$
$$+V_c \cdot (\mathrm{kAX} \cdot [\mathrm{Lext}] \cdot [\mathrm{Pit9}])$$

$$\frac{\mathrm{d}([\mathrm{Lend}] \cdot V_c)}{\mathrm{d}t} = +V_c \cdot (\mathrm{kE} \cdot [\mathrm{Pit1}])$$
$$+2 \cdot V_c \cdot (\mathrm{kE} \cdot [\mathrm{Pit2}])$$
$$+3 \cdot V_c \cdot (\mathrm{kE} \cdot [\mathrm{Pit3}])$$
$$+4 \cdot V_c \cdot (\mathrm{kE} \cdot [\mathrm{Pit4}])$$
$$+5 \cdot V_c \cdot (\mathrm{kE} \cdot [\mathrm{Pit5}])$$
$$+6 \cdot V_c \cdot (\mathrm{kE} \cdot [\mathrm{Pit6}])$$
$$+7 \cdot V_c \cdot (\mathrm{kE} \cdot [\mathrm{Pit7}])$$
$$+8 \cdot V_c \cdot (\mathrm{kE} \cdot [\mathrm{Pit8}])$$
$$+9 \cdot V_c \cdot (\mathrm{kE} \cdot [\mathrm{Pit9}])$$
$$+10 \cdot V_c \cdot (\mathrm{kE} \cdot [\mathrm{Pit10}])$$

$$\frac{\mathrm{d}([\mathrm{Nend}] \cdot V_c)}{\mathrm{d}t} = +V_c \cdot (\mathrm{kE} \cdot [\mathrm{Pit1}])$$
$$+V_c \cdot (\mathrm{kE} \cdot [\mathrm{Pit2}])$$
$$+V_c \cdot (\mathrm{kE} \cdot [\mathrm{Pit3}])$$
$$+V_c \cdot (\mathrm{kE} \cdot [\mathrm{Pit4}])$$
$$+V_c \cdot (\mathrm{kE} \cdot [\mathrm{Pit5}])$$
$$+V_c \cdot (\mathrm{kE} \cdot [\mathrm{Pit6}])$$
$$+V_c \cdot (\mathrm{kE} \cdot [\mathrm{Pit7}])$$
$$+V_c \cdot (\mathrm{kE} \cdot [\mathrm{Pit8}])$$
$$+V_c \cdot (\mathrm{kE} \cdot [\mathrm{Pit9}])$$
$$+V_c \cdot (\mathrm{kE} \cdot [\mathrm{Pit10}])$$

$$\frac{\mathrm{d}([\mathrm{Neff}]\cdot V_c)}{\mathrm{d}t} = +V_c\cdot(\mathrm{kL}\cdot[\mathrm{End1}]) +V_c\cdot(\mathrm{kL}\cdot[\mathrm{End2}]) +V_c\cdot(\mathrm{kL}\cdot[\mathrm{End3}]) +V_c\cdot(\mathrm{kL}\cdot[\mathrm{End4}]) +V_c\cdot(\mathrm{kL}\cdot[\mathrm{End5}]) +V_c\cdot(\mathrm{kL}\cdot[\mathrm{End6}]) +V_c\cdot(\mathrm{kL}\cdot[\mathrm{End7}]) +V_c\cdot(\mathrm{kL}\cdot[\mathrm{End8}]) +V_c\cdot(\mathrm{kL}\cdot[\mathrm{End9}]) +V_c\cdot(\mathrm{kL}\cdot[\mathrm{End10}])$$

$$\frac{\mathrm{d}([\mathrm{m0}]\cdot V_c)}{\mathrm{d}t} = +350\cdot V_c\cdot(\mathrm{kU}\cdot[\mathrm{Lint}])$$

$$\frac{\mathrm{d}([\mathrm{NPit}]\cdot V_c)}{\mathrm{d}t} = +V_c\cdot(\mathrm{kAX}\cdot[\mathrm{Lext}])$$

$$[\mathrm{LCX}] = \mathrm{Pit1.ParticleNumber} + 2\cdot\mathrm{Pit2.ParticleNumber} + 3\cdot\mathrm{Pit3.ParticleNumber} +4\cdot\mathrm{Pit4.ParticleNumber} + 5\cdot\mathrm{Pit5.ParticleNumber} + 6\cdot\mathrm{Pit6.ParticleNumber} +7\cdot\mathrm{Pit7.ParticleNumber} + 8\cdot\mathrm{Pit8.ParticleNumber} + 9\cdot\mathrm{Pit9.ParticleNumber} +10\cdot\mathrm{Pit10.ParticleNumber}$$

$$\mathrm{LendX} = \mathrm{End1.ParticleNumber} + 2\cdot\mathrm{End2.ParticleNumber} + 3\cdot\mathrm{End3.ParticleNumber} +4\cdot\mathrm{End4.ParticleNumber} + 5\cdot\mathrm{End5.ParticleNumber} + 6\cdot\mathrm{End6.ParticleNumber} +7\cdot\mathrm{End7.ParticleNumber} + 8\cdot\mathrm{End8.ParticleNumber} + 9\cdot\mathrm{End9.ParticleNumber} +10\cdot\mathrm{End10.ParticleNumber}$$

$$\mathrm{NendX} = \mathrm{End1.ParticleNumber} + \mathrm{End2.ParticleNumber} + \mathrm{End3.ParticleNumber} +\mathrm{End4.ParticleNumber} + \mathrm{End5.ParticleNumber} + \mathrm{End6.ParticleNumber} +\mathrm{End7.ParticleNumber} + \mathrm{End8.ParticleNumber} + \mathrm{End9.ParticleNumber} +\mathrm{End10.ParticleNumber}$$

$$\mathrm{NPitX} = \mathrm{Pit1.ParticleNumber} + \mathrm{Pit2.ParticleNumber} + \mathrm{Pit3.ParticleNumber} +\mathrm{Pit4.ParticleNumber} + \mathrm{Pit5.ParticleNumber} + \mathrm{Pit6.ParticleNumber} +\mathrm{Pit7.ParticleNumber} + \mathrm{Pit8.ParticleNumber} + \mathrm{Pit9.ParticleNumber} +\mathrm{Pit10.ParticleNumber}$$

$$\mathrm{kAX} = \frac{\mathrm{kA}}{\mathrm{NPitX}+1}$$

$$\mathrm{kW} = \begin{cases} \mathrm{Time} < \mathrm{iT} & 0 \\ 1e20 & \mathrm{else} \end{cases}$$

$$\mathrm{Leff} = \begin{cases} \mathrm{Nend.ParticleNumber} > 0 & \frac{\mathrm{Lend.ParticleNumber}}{\mathrm{Nend.ParticleNumber}} \\ 0 & \mathrm{else} \end{cases}$$

$$\mathrm{LeffX} = \begin{cases} \mathrm{NendX} > 0 & \frac{\mathrm{LendX}}{\mathrm{NendX}} \\ 0 & \mathrm{else} \end{cases}$$
