# Supplementary material for: Multi-Level Kinetic Model of mRNA Delivery via Transfection of Lipoplexes
Source: PLoS One. 2014 Sep 19;9(9):e107148. doi: 10.1371/journal.pone.0107148 (PMC4169511; doi:10.1371/journal.pone.0107148)
Supplement: File S1 — Code S1. Script for automated simulation of dose-response relationship. Code S2. script for automated simulation of lipoplex size dependency. Code S3. C# source code for program to set parameters in Copasi model. Code S4. C# source code for program to run Copasi model multiple times and analyze results in Igor Pro. Code S5. C# source code for program to run TFC.exe and optimize via simulated annealing algorithm. Code S6. C# source code for program to run Copasi streamlined (reduced) model multiple times and analyze results in Igor Pro. Code S7. C# source code for program to run TFRC.exe and optimize via simulated annealing algorithm. Code S8. Igor Pro procedure for analyzing results of Copasi model (TFC.cps and TFC.cps). Code S9. Perl script for running SPiM model. Code S10. Igor Pro procedure for analyzing results of SPiM model. Code S11. Igor Pro procedure for creating figures. Dataset S1. Dose-response data ( Figure 6 ). Dataset S2. GFP data ( Figure 4 ). Dataset S3. Lipoplex size data ( Figure 7 ). Dataset S4. Max GFP experiment ( Figure 5B ). Dataset S5Max GFP reduced model ( Figure 5B ). Dataset S6. Max GFP ( Figure 5B ). Dataset S7. Onset time experiment ( Figure 5A ). Dataset S8. Onset time reduced model ( Figure 5A ). Dataset S9. Onset time ( Figure 5A ). Dataset S10. Time courses ( Figure 3 ). Model S1. Copasi model for deterministic simulation of multiple lipoplex model. Model S2. SBML model for deterministic simulation of multiple lipoplex model. Model S3. Copasi model for stochastic simulation of multiple lipoplex model. Model S4. SBML model for stochastic simulation of multiple lipoplex model. Model S5. Copasi model for deterministic simulation of streamlined (reduced) model. Model S6. SBML model for deterministic simulation of streamlined (reduced) model. Model S7. Copasi model for stochastic simulation of streamlined (reduced) model. Model S8. SBML model for stochastic simulation of streamlined (reduced) model. Model S9. SPiM model. Model S10. Versio [file pone.0107148.s001.zip › TextS001.doc]

# Multi-Level Kinetic Model of mRNA Delivery via Transfection of Lipoplexes - Supplementary Information

Thomas S. Ligon, Carolin Leonhardt, Joachim O. Rädler

## File Names

As part of the publication process, many file names have been changed. The following table shows how they were changed, because the original name is often used in scripts and programs, so it might be useful.

| **new name** | **original name** |
| --- | --- |
| CodeS001.cmd | TFCDRI.cmd |
| CodeS002.cmd | TFCLSize.cmd |
| CodeS003.cs | SetCopasiParams.cs |
| CodeS004.cs | TFC.cs |
| CodeS005.cs | TFCSA.cs |
| CodeS006.cs | TFRC.cs |
| CodeS007.cs | TFRCSA.cs |
| CodeS008.ips | TFC.ipf |
| CodeS009.pl | mRNARunnerTFS.pl |
| CodeS010.ipf | TFS.ipf |
| CodeS011.ipf | TFFig.ipf |
| DatasetS001.txt | TFFig.DR.txt |
| DatasetS002.txt | TFFig.GFP.txt |
| DatasetS003.txt | TFFig.LSize.txt |
| DatasetS004.txt | TFFig.maxGFP.Exp.txt |
| DatasetS005.txt | TFFig.maxGFP.R.txt |
| DatasetS006.txt | TFFig.maxGFP.txt |
| DatasetS007.txt | TFFig.t0.Exp.txt |
| DatasetS008.txt | TFFig.t0.R.txt |
| DatasetS009.txt | TFFig.t0.txt |
| DatasetS010.txt | TFFig.TimeCourses.txt |
| ModelS001.cps | TFD.cps |
| ModelS002.xml | TFD.xml |
| ModelS003.cps | TFS.cps |
| ModelS004.xml | TFS.xml |
| ModelS005.cps | TFRD.cps |
| ModelS006.xml | TFRD.xml |
| ModelS007.cps | TFRS.cps |
| ModelS008.xml | TFRS.xml |
| ModelS009.spi | TF.spi |
| ModelS010.bngl | TF1.bngl |
| ModelS011.xml | TF1.xml |
| TextS001.doc | TF-SI-2014-07-15.doc |
| TextS002.tex | ODEs.Copasi.tex |
| TextS003.tex | ODEs.tex |
| TextS004.pdf | ODEs.pdf |

## Model Description

### Species and Pseudo Species

The model also contains a number of “species” that do not correspond to biological entities. For example, LC appears as a product in all “attach reactions”, but does not appear anywhere as a reactant. As a result, it is simply a convenient way to count the cumulative number of lipoplexes that have attached to the cell surface, making it unnecessary to count this number by analyzing the time-course files after running the simulation. Species and pseudo species are listed in Table 2.

| **A Species** | |
| --- | --- |
| **Name** | **Description** |
| Lext | External lipoplex. |
| Pit_i_ | Clathrin-coated pit containing i lipoplexes. |
| End_i_ | Endosome containing i lipoplexes. |
| Lint | Internal lipoplex. |
| mRNA | mRNA molecules. |
| GFP | GFP molecules. |
| **B Pseudo Species** | |
| **Name** | **Description** |
| LC | number of lipoplexes on the cell surface (cumulative) |
| LCX | number of lipoplexes on the cell surface (transient) |
| Npit | number of clathrin-coated pits (cumulative) |
| Lend | number of lipoplexes contained in endosomes (transient) |
| Nend | number of endosomes (cumulative) |
| Neff | number of lysed endosomes (cumulative) |
| m0 | number of mRNA molecules (cumulative) |

Table 2. Species and Pseudo Species. A) Species. B) Pseudo species.

### Reactions

Reactions, in the style of chemical reactions, are the standard way to describe a model in SBML. Copasi also converts these reactions into ODEs. The reactions are described in “Results and Discussion” and documented in Table 3.

| **Name** | **Reaction** | **Description** |
| --- | --- | --- |
| wash | Lext -> | wash cells, removing remaining lipoplexes |
| attach_0_ | Lext -> Pit_1_ + LC + NPit | attach lipoplex to cell surface |
| attach_i_ | Lext + Pit_i_ -> Pit_i+1_ + LC |  |
| endo_i_ | Pit_i_ -> End_i_ + i*Lend + Nend | endocytosis, converting pit to endosome |
| lyse_i_ | End_i_ -> i*Lint + Neff | lysis of endosome |
| degE_i_ | End_i_ -> | degradation of endosome |
| unpack | Lint -> 350 * mRNA + 350 * m0 | upacking of lipoplexes, releasing mRNA |
| degL | Lint -> | degradation of lipoplex |
| translate | mRNA -> mRNA + GFP | translation |
| degM | mRNA -> | degradation of mRNA |
| degG | GFP -> | degradation of GFP |

Table 3. Reactions.

### Reaction Rates

See Table 1 in main text.

### Literature values

#### Delivery parameters

|  | **Varga 2005** | **Zhou 2007** | **Dinh 2007** |
| --- | --- | --- | --- |
| kA | k_bind_uptake_ = .12-.36 h^-1^ | k_MP_ = .006-.35 h^-1^ | k_int_ = .5 h^-1^ |
| kE |  | k_PE_ = .16-.5 h^-1^ |  |
| kL | k_escape_ = .003-.96 h^-1^ | k_EC_ = .001-90 h^-1^ | k_escape_ = .05-.2 h^-1^ |
| kU | k_unpack_ = .6-30 h^-1^ |  | k_unpack_ = .02-.05 h-1 |

, ,

#### Maturation

| **Author** | **time(min)** | **GFP** | **organism** | **comments** |
| --- | --- | --- | --- | --- |
| Kremers (2007) | 89 128 | EGFP, SGFP1 | bacteria and mammalian cells | 128 min |
| Megerle (2008) | 6.5 |  | E. coli |  |
| Reid (1997) | 77 | S65T-GFP | inclusion bodies of E. coli |  |
| Sniegowski (2005) | 41 77(EGFP) |  | E. coli |  |
| Tsien (1998) | 30-120 | GFP | E. coli | oxidation: 120 min for WT GFP, 30 min for S65T mutant |

Note that time is sometimes reported as the reciprocal of the rate, as in Megerle 2008, or as ln(2) times this value, in order to represent half-life. The literature values reported here are half-lives.

Comments

Kremers (2007)

EGFP: K_ox_ = 1.3*10^-4^*s^-1^ = 128 min

SGFP1: K_ox_ = 5.1*10^-4^*s^-1^ = 33 min

Reid (1997): oxidation is 1.51*10^-4^ s^-1^ = 9*10-3 m^-1^ = .54 h^-1^

Therefore, the reciprocal rate constant for ibS65T-GFP fluorescence acquisition (1/kib) is equal to the sum of the reciprocal rate constants for each step in chromophore formation (1/*k*f + 1/*k*c + 1/*k*ox).

That gives .49 h^-1^

Sniegowski (2005): 1.28h at pH7 for EGFP (Table I). 1.28*60=77min.

### ODEs

The ordinary differential equations are equivalent to the reactions as described above, but the reactions are the typical formulation of the model in stochastic simulations, and the ODEs are the typical formulation in deterministic simulations. Note that equations (1)-(11) refer to species, while (12)-(22) refer to pseudo species, as defined above. The pseudo species do not affect the model, but are used as counters to simplify the analysis. [X] refers to the concentration of X, and |X| refers to the number of particles of X contained in the default compartment.

$\frac{d\left[ \text{Lext} \right]}{\text{dt}}=-\text{kAX}*\left[ \text{Lext} \right]-\sum\text{kAX}*\left[ \text{Lext} \right]*\left[ \text{Pit}_{i} \right]-\text{kW}*\left[ \text{Lext} \right]$	(1)

$\frac{d\left[ \text{Pit}_{i} \right]}{\text{dt}}\text{=+}\text{kAX}*\left[ \text{Lext} \right]-\text{kAX}*\left[ \text{Lext} \right]*\left[ \text{Pit}_{i} \right]-\text{kE}*\left[ \text{Pit}_{i} \right]$	(2)

$\begin{aligned} \frac{d\left[ \text{Pit}_{i+1} \right]}{\text{dt}}\text{=+}\text{kAX}*\left[ \text{Lext} \right]*\left[ \text{Pit}_{i} \right]-\text{kAX}*\left[ \text{Lext} \right]*\left[ \text{Pit}_{i+1} \right]-\text{kE}*\left[ \text{Pit}_{i+1} \right] \\ i=1\text{.}\text{.}\text{.}9 \end{aligned}$	(3)

$\begin{aligned} \frac{d\left[ \text{End}_{i} \right]}{\text{dt}}\text{=+}\text{kE}*\left[ \text{Pit}_{i} \right]-\text{kL}\cdot\left[ \text{End}_{i} \right]-\text{dE}\left[ \text{End}_{i} \right] \\ i=1\text{.}\text{.}\text{.}\text{10} \end{aligned}$	(4)

$\frac{d\left[ \text{Lin} \right]}{\text{dt}}\text{=+}\sum i*\text{kL}*\left[ \text{End}_{i} \right]-\text{kU}*\left[ \text{Lin} \right]-\text{dL}*\left[ \text{Lin} \right]$	(5)

$\frac{d\left[ \text{mRNA} \right]}{\text{dt}}\text{=+}\text{350}*\text{kU}*\left[ \text{Lin} \right]-\text{dM}*\left[ \text{mRNA} \right]$	(6)

$\frac{d\left[ \text{GFPU} \right]}{\text{dt}}\text{=+}\text{kTL}*\left[ \text{mRNA} \right]-\text{dG}*\left[ \text{GFPU} \right]$	(7)

$\frac{d\left[ \text{GFP} \right]}{\text{dt}}\text{=+}\text{kM}*\left[ \text{GFPU} \right]-\text{dG}*\left[ \text{GFP} \right]$	(8)

$\begin{aligned} \text{NPitX}\text{=+}\sum\left| \text{Pit}_{i} \right| \\ \left| \text{Pit}_{i} \right|=\text{particles} \end{aligned}$	(9)

$\text{kAX}=\frac{\text{kA}}{\text{NPitX}+1}$	(10)

$\text{kW}=\text{if}(\text{Time}<\text{iT})\text{then}(0)\text{else}(1e\text{20})$	(11)

$\frac{d\left[ \text{LC} \right]}{\text{dt}}\text{=+}\text{kAX}*\left[ \text{Lext} \right]+\sum\text{kAX}*\left[ \text{Lext} \right]*\left[ \text{Pit}_{i} \right]$	(12)

$\frac{d\left[ \text{Lend} \right]}{\text{dt}}\text{=+}\sum i*\text{kE}*\left[ \text{Pit}_{i} \right]$	(13)

$\frac{d\left[ \text{Nend} \right]}{\text{dt}}\text{=+}\sum\text{kE}*\left[ \text{Pit}_{i} \right]$	(14)

$\frac{d\left[ \text{Neff} \right]}{\text{dt}}\text{=+}\sum\text{kL}*\left[ \text{End}_{i} \right]$	(15)

$\frac{d\left[ m0 \right]}{\text{dt}}\text{=+}\text{350}*\text{kU}*\left[ \text{Lin} \right]$	(16)

$\frac{d\left[ \text{NPit} \right]}{\text{dt}}=\text{kAX}*\left[ \text{Lext} \right]$	(17)

$\left[ \text{LCX} \right]\text{=+}\sum i*\left| \text{Pit}_{i} \right|$	(18)

$\text{LendX}\text{=+}\sum i*\left| \text{End}_{i} \right|$	(19)

$\text{NendX}\text{=+}\sum\left| \text{End}_{i} \right|$	(20)

$\text{Leff}=\text{if}(\left| \text{Nend} \right|>0)\text{then}(\frac{\left| \text{Lend} \right|}{\left| \text{Nend} \right|})\text{else}(0)$	(21)

$\text{LeffX}=\text{if}(\text{NendX}>0)\text{then}(\frac{\text{LendX}}{\text{NendX}})\text{else}(0)$	(22)

### Pi Calculus Model

A full understanding of this implementation of the model requires knowledge of Pi Calculus, and we won’t repeat that here, but nevertheless explain enough of the logic to make the code fairly readable for people who do not know Pi Calculus.

The Pi Calculus implementation of the model, which was run in SPiM, deals with the variable lipoplex size by including the size as a parameter in the process. In fact, the process Lext has one parameter for the number of mRNA molecules and one parameter for the time until the cells are washed. The Lext process is initialized for a limited number of 11 possible sizes in the “run” statements near the end of the code.

run 6 of Lext(347,0.0)

This would benefit greatly from the ability to initialize the process using a function. SPiM runs stochastic simulations of processes, but we are often using parameters of processes, so our reactions need to include the reaction rate explicitly as part of a “delay” statement. We have also used explicit lists of 10 possible sizes for pits and endosomes. In Pi Calculus, we could have used parameters to avoid this, but this would have required writing 10 levels of nested “if” statements in order to formulate the reactions on the parameters, increasing the complexity by more than we would have gained by avoiding the lists. The reaction “Lint(n)->n*mRNA()” cannot be written with a variable n, so we have use a series of processes called Count512() etc.

Here is an example of the wash and attach reactions in Pi Calculus. External lipoplexes, Lext(n,t), contain n mRNA molecules and time t remaining until being washed. The Lext process sends a message on the attach channel (!attach), which is received by one of the pit processes (?attach), for example Pit0, which then creates a pit of size 1 (Pit1) containing n mRNA molecules. The wash reaction uses a delay statement instead of a channel, because it involves the parameter of a single process as opposed to two different processes. Note that comments are enclosed in (* and *). This code implements the timer-based wash reaction and the attach_0_ reaction. (In contrast with the SBML implementation, the Pi Calculus implementation contains an explicit Pit_0_.) Each action is followed by a semicolon-separated list of processes that run after the action completes. For example, within the Lext() process, if the timer reaches the value of iT (the else branch), the process continues as (), i.e. nothing. Within the Pit0() process, if the ?attach(n) channel is activated, the process continues as a Pit1(n), and Pit0(), and the 2 pseudo species NPit() and LC(), which are just counters.

wash: Lext ->

attach_0_: Lext -> Pit_1_ + LC + NPit

```
...
let Lext(n:int,t:float) =
  if (t<iT) then
    do !attach(n) (* attach to cell surface *)
    or delay@1000.0;Lext(n,t+0.001) (* increment timer after 0.001 h *)
  else
    () (* external lipoplexes are washed away at 1 hour *)
and Pit0() =
  ?attach(n); Pit1(n); Pit0(); NPit(); LC()
...
```

This implementation of the model runs in a version of SPiM that was released in 2007 and supports automation via command-line invocation. The newer version of SPiM, released as a beta in 2011, includes a new method for creating plots, but that runs into an unsurmountable performance problem when using parameters of processes. This is an example of our observation that we can move the burden of the combinatorial explosion from the user to the tool, but then the tool needs to deal with dynamic creation of species, with all consequences for performance.

In summary, the use of parameters of processes in SPiM makes it possible to dynamically create “species” instead of listing them, thus solving the combinatorial explosion for the user, but this leads to various limitations that would need to be resolved before the technique could be used more extensively.

```
directive sample 30.0 10000
directive plot LC();NPit();Lend();Nend();Neff();Lint();m0();mRNA();GFP();
    Pit1();Pit2();Pit3();Pit4();Pit5();Pit6();Pit7();Pit8();Pit9();Pit10();
    End1();End2();End3();End4();End5();End6();End7();End8();End9();End10()

(* definition of constants *)
val infDelay = 0.000000001
val iT = 1.0 (* incubation time *)
val kA = 0.03 (* attach *)
val kE = 0.45 (* endocytosis *)
val kL = 0.025 (* lyse endosome *)
val kU = 1000000000.0 (* unpack lipoplex *)
val kTL = 100.0 (* translate *)
val dM = 0.051 (* delta = mRNA degradation *)
val dG = 0.056 (* beta = GFP degradation *)
val dL = 0.000000001 (* lipoplex degradation *)
val dE = 0.01 (* endosome degradation *)

new attach@kA:chan(int)

let Lext(n:int,t:float) =
  if (t<iT) then
    do !attach(n) (* attach to cell surface *)
    or delay@1000.0;Lext(n,t+0.001) (* increment timer after 0.001 h *)
  else
    () (* external lipoplexes are washed away at 1 hour *)
and Pit0() =
  ?attach(n); Pit1(n); Pit0(); NPit(); LC()
and Pit1(n1:int) =
  do ?attach(n); Pit2(n1,n); LC()
  or delay@kE; (End1(n1) | Lend() | Nend())
and Pit2(n1:int,n2:int) =
  do ?attach(n); Pit3(n1,n2,n); LC()
  or delay@kE; (End2(n1,n2) | 2 of Lend() | Nend())
and Pit3(n1:int,n2:int,n3:int) =
  do ?attach(n); Pit4(n1,n2,n3,n); LC()
  or delay@kE; (End3(n1,n2,n3) | 3 of Lend() | Nend())
and Pit4(n1:int,n2:int,n3:int,n4:int) =
  do ?attach(n); Pit5(n1,n2,n3,n4,n); LC()
  or delay@kE; (End4(n1,n2,n3,n4) | 4 of Lend() | Nend())
and Pit5(n1:int,n2:int,n3:int,n4:int,n5:int) =
  do ?attach(n); Pit6(n1,n2,n3,n4,n5,n); LC()
  or delay@kE; (End5(n1,n2,n3,n4,n5) | 5 of Lend() | Nend())
and Pit6(n1:int,n2:int,n3:int,n4:int,n5:int,n6:int) =
  do ?attach(n); Pit7(n1,n2,n3,n4,n5,n6,n); LC()
  or delay@kE; (End6(n1,n2,n3,n4,n5,n6) | 6 of Lend() | Nend())
and Pit7(n1:int,n2:int,n3:int,n4:int,n5:int,n6:int,n7:int) =
  do ?attach(n); Pit8(n1,n2,n3,n4,n5,n6,n7,n); LC()
  or delay@kE; (End7(n1,n2,n3,n4,n5,n6,n7) | 7 of Lend() | Nend())
and Pit8(n1:int,n2:int,n3:int,n4:int,n5:int,n6:int,n7:int,n8:int) =
  do ?attach(n); Pit9(n1,n2,n3,n4,n5,n6,n7,n8,n); LC()
  or delay@kE; (End8(n1,n2,n3,n4,n5,n6,n7,n8) | 8 of Lend() | Nend())
and Pit9(n1:int,n2:int,n3:int,n4:int,n5:int,n6:int,n7:int,n8:int,n9:int) =
  do ?attach(n); Pit10(n1,n2,n3,n4,n5,n6,n7,n8,n9,n); LC()
  or delay@kE; (End9(n1,n2,n3,n4,n5,n6,n7,n8,n9) | 9 of Lend() | Nend())
and Pit10(n1:int,n2:int,n3:int,n4:int,n5:int,n6:int,n7:int,n8:int,n9:int,n10:int) =
  delay@kE; (End10(n1,n2,n3,n4,n5,n6,n7,n8,n9,n10) | 10 of Lend() | Nend())
and End1(n1:int) =
  do delay@kL;(Lint(n1) | Neff())
  or delay@dE; ()
and End2(n1:int,n2:int) =
  do delay@kL;(Lint(n1) | Lint(n2) | Neff())
  or delay@dE; ()
and End3(n1:int,n2:int,n3:int) =
  do delay@kL;(Lint(n1) | Lint(n2) | Lint(n3) | Neff())
  or delay@dE; ()
and End4(n1:int,n2:int,n3:int,n4:int) =
  do delay@kL;(Lint(n1) | Lint(n2) | Lint(n3) | Lint(n4) | Neff())
  or delay@dE; ()
and End5(n1:int,n2:int,n3:int,n4:int,n5:int) =
  do delay@kL;(Lint(n1) | Lint(n2) | Lint(n3) | Lint(n4) | Lint(n5) | Neff())
  or delay@dE; ()
and End6(n1:int,n2:int,n3:int,n4:int,n5:int,n6:int) =
  do delay@kL;(Lint(n1) | Lint(n2) | Lint(n3) | Lint(n4) | Lint(n5) | Lint(n6) | Neff())
  or delay@dE; ()
and End7(n1:int,n2:int,n3:int,n4:int,n5:int,n6:int,n7:int) =
  do delay@kL;(Lint(n1) | Lint(n2) | Lint(n3) | Lint(n4) | Lint(n5) | Lint(n6) | Lint(n7) | Neff())
  or delay@dE; ()
and End8(n1:int,n2:int,n3:int,n4:int,n5:int,n6:int,n7:int,n8:int) =
  do delay@kL;(Lint(n1) | Lint(n2) | Lint(n3) | Lint(n4) | Lint(n5) | Lint(n6) | Lint(n7) |
    Lint(n8) | Neff())
  or delay@dE; ()
and End9(n1:int,n2:int,n3:int,n4:int,n5:int,n6:int,n7:int,n8:int,n9:int) =
  do delay@kL;(Lint(n1) | Lint(n2) | Lint(n3) | Lint(n4) | Lint(n5) | Lint(n6) | Lint(n7) |
    Lint(n8) | Lint(n9) | Neff())
  or delay@dE; ()
and End10(n1:int,n2:int,n3:int,n4:int,n5:int,n6:int,n7:int,n8:int,n9:int,n10:int) =
  do delay@kL;(Lint(n1) | Lint(n2) | Lint(n3) | Lint(n4) | Lint(n5) | Lint(n6) | Lint(n7) |
    Lint(n8) | Lint(n9) | Lint(n10) | Neff())
  or delay@dE; ()
and Lint(n:int) =
  do delay@kU;Count512(n) (* lipoplex unpacks, create n of mRNA() *)
  or delay@dL; () (* lipoplex degrades *)
and Count512(n:int) =
  if n>=512 then (512 of mRNA() | 512 of m0() | Count256(n-512))
  else Count256(n)
and Count256(n:int) =
  if n>=256 then (256 of mRNA() | 256 of m0() | Count128(n-256))
  else Count128(n)
and Count128(n:int) =
  if n>=128 then (128 of mRNA() | 128 of m0() | Count64(n-128))
  else Count64(n)
and Count64(n:int) =
  if n>=64 then (64 of mRNA() | 64 of m0() | Count32(n-64))
  else Count32(n)
and Count32(n:int) =
  if n>=32 then (32 of mRNA() | 32 of m0() | Count16(n-32))
  else Count16(n)
and Count16(n:int) =
  if n>=16 then (16 of mRNA() | 16 of m0() | Count8(n-16))
  else Count8(n)
and Count8(n:int) =
  if n>=8 then (8 of mRNA() | 8 of m0() | Count4(n-8))
  else Count4(n)
and Count4(n:int) =
  if n>=4 then (4 of mRNA() | 4 of m0() | Count2(n-4))
  else Count2(n)
and Count2(n:int) =
  if n>=2 then (2 of mRNA() | 2 of m0() | Count1(n-2))
  else Count1(n)
and Count1(n:int) =
  if n>=1 then (1 of mRNA() | 1 of m0())
and mRNA() = do delay@kTL;(mRNA() | GFP()) (* translation *)
             or delay@dM;() (* mRNA degradation *)
and GFP() = delay@dG;() (* GFP degradation *)
and LC() = delay@infDelay;()
and NPit() = delay@infDelay;()
and Lend() = delay@infDelay;()
and Nend() = delay@infDelay;()
and Neff() = delay@infDelay;()
and m0() = delay@infDelay;()

run 1 of Lext(345,0.0)
run 3 of Lext(346,0.0)
run 6 of Lext(347,0.0)
run 12 of Lext(348,0.0)
run 18 of Lext(349,0.0)
run 20 of Lext(350,0.0)
run 18 of Lext(351,0.0)
run 12 of Lext(352,0.0)
run 6 of Lext(353,0.0)
run 3 of Lext(354,0.0)
run 1 of Lext(355,0.0)
(* run 100 of Lext(350,0.0) *)
run 1 of Pit0()
```

### BNGL Model

A full understanding of this implementation of the model requires knowledge of BNGL, and we won’t repeat that here, but nevertheless explain enough of the logic to make the code fairly readable for people who do not know BNGL.

Here is an example of a basic reaction, where an external lipoplex attaches to an existing pit of size 1 to form a new pit of size 2. It could be documented as

Lext(n2) + Pit_1_(n1) -> Pit_2_(n1,n2)

The BNGL code can be understood as: A free external lipoplex and a lipoplex bound to a pit (this is p1!1 and l1!1) react to form 2 lipoplexes bound to a pit (p!1 and l1!1 plus p!2 and l2!2). The reaction rate is kA.

Lext(p,n) + Lext(p!1,n).Pit(s~p,l1!1,l2,l3,l4,l5,l6,l7,l8,l9,l10) -> Lext(p!2,n).Lext(p!1,n).Pit(s~p,l1!1,l2!2,l3,l4,l5,l6,l7,l8,l9,l10) kA

This is a prototype of an implementation of the model written in BNGL and run in NFSim . In these tools, we can easily write the attributed species Lint(n), but there is no way to use the variable n in the reaction without explicitly fixing its value in the reactant, so it does not currently solve our modeling problem. This is the kind of limitation preventing us from creating an NFsim model that reproduces the other two versions. We also encountered problems very similar to those we observed in SPiM, including the difficulty in initializing the external lipoplexes Lext, and in writing the reaction Lint(n)->n*mRNA().

In summary, the family of “rules based” tools using languages such as BNGL contains methods for creating variability of species and targeted at modeling complex protein reactions. This looks very promising for our model, but some key parts, such as the ability to use variable parameters in reactions, are missing.

#line beginning with a hash symbol are comments

#definition of constants

```
begin parameters
  kFast 1e20
  kA 0.016*3600 # attach
  kE 0.45*3600 # endocytosis
  kL 0.4*3600 # lyse endosome
  kU 0.4*3600 # unpack lipoplex
  kTL 100.0*3600 # translate
  dW 0 # wash (degrade external lipoplexes)
  dM 0.051*3600 # delta mRNA degradation
  dG 0.056*3600 # beta GFP degradation
  dL 0.01*3600 # lipoplex degradation
  dE 1.0*3600 # endosome degradation
end parameters

#definition of species
begin molecule types
  #Lext(p,n~345~355)
  #Lext(p,n~345~355~PLUS~MINUS)
  #note that it we specify all possible value of n,
  #because we ran into limitations with PLUS MINUS
  Lext(p,n~345~346~347~348~349~350~351~352~353~354~355)
  #a pit can be of type pit (s~p) or endosome (s~e),
  #because this is the only way we were able to implement the endocytosis,
  #which needs to conserve the number of mRNA molecules from pit to endosome
  Pit(s~p~e,l1,l2,l3,l4,l5,l6,l7,l8,l9,l10) # pit now has flag for pit vs. endosome
  #End(l1,l2,l3,l4,l5,l6,l7,l8,l9,l10)
  Lint(n~345~346~347~348~349~350~351~352~353~354~355)
  mRNA population
  GFP population
  Trash
  I population
  Timer population
end molecule types

#this is the initialization of the species
#as in Pi Calculus, we would theoretically need to list 175 values for Lext
begin seed species
  Lext(p,n~345) 1
  Lext(p,n~346) 2
  Lext(p,n~347) 6
  Lext(p,n~348) 12
  Lext(p,n~349) 18
  Lext(p,n~350) 20
  Lext(p,n~351) 18
  Lext(p,n~352) 12
  Lext(p,n~353) 6
  Lext(p,n~354) 3
  Lext(p,n~355) 1
  Pit(s~p,l1,l2,l3,l4,l5,l6,l7,l8,l9,l10) 1
  mRNA 0
  GFP 0
  I 1
  Timer 0
end seed species

begin observables
  Molecules Lext Lext(p,n)
  Molecules Pit Pit(s~p)
  Molecules Endo Pit(s~e)
  #Molecules Pit0 Pit()
  #Molecules Pit1 Lext(p!1,n).Pit(l1!1,l2,l3,l4,l5,l6,l7,l8,l9,l10)
  #Molecules Pit2 Lext(p!2,n).Lext(p!1,n).Pit(l1!1,l2!2,l3,l4,l5,l6,l7,l8,l9,l10)
  #Molecules Pit3 Lext(p!3,n).Lext(p!2,n).Lext(p!1,n).Pit(l1!1,l2!2,l3!3,l4,l5,l6,l7,l8,l9,l10)
  #Molecules Pit4 Lext(p!4,n).Lext(p!3,n).Lext(p!2,n).Lext(p!1,n).Pit(l1!1,l2!2,l3!3,l4!4,l5,l6,l7,l8,l9,l10)
  #Molecules Pit5 Lext(p!5,n).Lext(p!4,n).Lext(p!3,n).Lext(p!2,n).Lext(p!1,n).Pit(l1!1,l2!2,l3!3,l4!4,l5!5,l6,l7,l8,l9,l10)
  #Molecules Pit6 Lext(p!6,n).Lext(p!5,n).Lext(p!4,n).Lext(p!3,n).Lext(p!2,n).Lext(p!1,n).Pit(l1!1,l2!2,l3!3,l4!4,l5!5,l6!6,l7,l8,l9,l10)
  #Molecules Pit7 Lext(p!7,n).Lext(p!6,n).Lext(p!5,n).Lext(p!4,n).Lext(p!3,n).Lext(p!2,n).Lext(p!1,n).Pit(l1!1,l2!2,l3!3,l4!4,l5!5,l6!6,l7!7,l8,l9,l10)
  #Molecules Pit8 Lext(p!8,n).Lext(p!7,n).Lext(p!6,n).Lext(p!5,n).Lext(p!4,n).Lext(p!3,n).Lext(p!2,n).Lext(p!1,n).Pit(l1!1,l2!2,l3!3,l4!4,l5!5,l6!6,l7!7,l8!8,l9,l10)
  #Molecules Pit9 Lext(p!9,n).Lext(p!8,n).Lext(p!7,n).Lext(p!6,n).Lext(p!5,n).Lext(p!4,n).Lext(p!3,n).Lext(p!2,n).Lext(p!1,n).Pit(l1!1,l2!2,l3!3,l4!4,l5!5,l6!6,l7!7,l8!8,l9!9,l10)
  #Molecules Pit10 Lext(p!10,n).Lext(p!9,n).Lext(p!8,n).Lext(p!7,n).Lext(p!6,n).Lext(p!5,n).Lext(p!4,n).Lext(p!3,n).Lext(p!2,n).Lext(p!1,n).Pit(l1!1,l2!2,l3!3,l4!4,l5!5,l6!6,l7!7,l8!8,l9!9,l10!10)
  Molecules Lint Lint()
  Molecules mRNA mRNA
  Molecules GFP GFP
  Molecules TimerCount Timer
end observables

begin functions
  wash = if(TimerCount>3600,1e20,0)
end functions

begin reaction rules
  # degrade external lipoplex (washing)
  Lext(p,n) -> Trash wash
  # attach - external lipoplex attaches to clathrin-coated pit
  Lext(p,n) + Pit(s~p,l1,l2,l3,l4,l5,l6,l7,l8,l9,l10) -> Lext(p!1,n).Pit(s~p,l1!1,l2,l3,l4,l5,l6,l7,l8,l9,l10) + Pit(s~p,l1,l2,l3,l4,l5,l6,l7,l8,l9,l10) kA
  Lext(p,n) + Lext(p!1,n).Pit(s~p,l1!1,l2,l3,l4,l5,l6,l7,l8,l9,l10) -> Lext(p!2,n).Lext(p!1,n).Pit(s~p,l1!1,l2!2,l3,l4,l5,l6,l7,l8,l9,l10) kA
  Lext(p,n) + Lext(p!2,n).Lext(p!1,n).Pit(s~p,l1!1,l2!2,l3,l4,l5,l6,l7,l8,l9,l10) -> Lext(p!3,n).Lext(p!2,n).Lext(p!1,n).Pit(s~p,l1!1,l2!2,l3!3,l4,l5,l6,l7,l8,l9,l10) kA
  Lext(p,n) + Lext(p!3,n).Lext(p!2,n).Lext(p!1,n).Pit(s~p,l1!1,l2!2,l3!3,l4,l5,l6,l7,l8,l9,l10) -> Lext(p!4,n).Lext(p!3,n).Lext(p!2,n).Lext(p!1,n).Pit(s~p,l1!1,l2!2,l3!3,l4!4,l5,l6,l7,l8,l9,l10) kA
  Lext(p,n) + Lext(p!4,n).Lext(p!3,n).Lext(p!2,n).Lext(p!1,n).Pit(s~p,l1!1,l2!2,l3!3,l4!4,l5,l6,l7,l8,l9,l10) -> Lext(p!5,n).Lext(p!4,n).Lext(p!3,n).Lext(p!2,n).Lext(p!1,n).Pit(s~p,l1!1,l2!2,l3!3,l4!4,l5!5,l6,l7,l8,l9,l10) kA
  Lext(p,n) + Lext(p!5,n).Lext(p!4,n).Lext(p!3,n).Lext(p!2,n).Lext(p!1,n).Pit(s~p,l1!1,l2!2,l3!3,l4!4,l5!5,l6,l7,l8,l9,l10) -> Lext(p!6,n).Lext(p!5,n).Lext(p!4,n).Lext(p!3,n).Lext(p!2,n).Lext(p!1,n).Pit(s~p,l1!1,l2!2,l3!3,l4!4,l5!5,l6!6,l7,l8,l9,l10) kA
  Lext(p,n) + Lext(p!6,n).Lext(p!5,n).Lext(p!4,n).Lext(p!3,n).Lext(p!2,n).Lext(p!1,n).Pit(s~p,l1!1,l2!2,l3!3,l4!4,l5!5,l6!6,l7,l8,l9,l10) -> \
    Lext(p!7,n).Lext(p!6,n).Lext(p!5,n).Lext(p!4,n).Lext(p!3,n).Lext(p!2,n).Lext(p!1,n).Pit(s~p,l1!1,l2!2,l3!3,l4!4,l5!5,l6!6,l7!7,l8,l9,l10) kA
  #Lext(p,n) + Lext(p!7,n).Lext(p!6,n).Lext(p!5,n).Lext(p!4,n).Lext(p!3,n).Lext(p!2,n).Lext(p!1,n).Pit(s~p,l1!1,l2!2,l3!3,l4!4,l5!5,l6!6,l7!7,l8,l9,l10) -> \
  #  Lext(p!8,n).Lext(p!7,n).Lext(p!6,n).Lext(p!5,n).Lext(p!4,n).Lext(p!3,n).Lext(p!2,n).Lext(p!1,n).Pit(s~p,l1!1,l2!2,l3!3,l4!4,l5!5,l6!6,l7!7,l8!8,l9,l10) kA
  #Lext(p,n) + Lext(p!8,n).Lext(p!7,n).Lext(p!6,n).Lext(p!5,n).Lext(p!4,n).Lext(p!3,n).Lext(p!2,n).Lext(p!1,n).Pit(s~p,l1!1,l2!2,l3!3,l4!4,l5!5,l6!6,l7!7,l8!8,l9,l10) -> \
  #  Lext(p!9,n).Lext(p!8,n).Lext(p!7,n).Lext(p!6,n).Lext(p!5,n).Lext(p!4,n).Lext(p!3,n).Lext(p!2,n).Lext(p!1,n).Pit(s~p,l1!1,l2!2,l3!3,l4!4,l5!5,l6!6,l7!7,l8!8,l9!9,l10) kA
  #Lext(p,n) + Lext(p!9,n).Lext(p!8,n).Lext(p!7,n).Lext(p!6,n).Lext(p!5,n).Lext(p!4,n).Lext(p!3,n).Lext(p!2,n).Lext(p!1,n).Pit(s~p,l1!1,l2!2,l3!3,l4!4,l5!5,l6!6,l7!7,l8!8,l9!9,l10) -> \
  #  Lext(p!10,n).Lext(p!9,n).Lext(p!8,n).Lext(p!7,n).Lext(p!6,n).Lext(p!5,n).Lext(p!4,n).Lext(p!3,n).Lext(p!2,n).Lext(p!1,n).Pit(s~p,l1!1,l2!2,l3!3,l4!4,l5!5,l6!6,l7!7,l8!8,l9!9,l10!10) kA
  # endocytosis - pit is converted to endosome
  Lext(p!1,n).Pit(s~p,l1!1,l2,l3,l4,l5,l6,l7,l8,l9,l10) -> Lext(p!1,n).Pit(s~e,l1!1,l2,l3,l4,l5,l6,l7,l8,l9,l10) kE DeleteMolecules
  Lext(p!2,n).Lext(p!1,n).Pit(s~p,l1!1,l2!2,l3,l4,l5,l6,l7,l8,l9,l10) -> Lext(p!2,n).Lext(p!1,n).Pit(s~e,l1!1,l2!2,l3,l4,l5,l6,l7,l8,l9,l10) kE DeleteMolecules
  Lext(p!3,n).Lext(p!2,n).Lext(p!1,n).Pit(s~p,l1!1,l2!2,l3!3,l4,l5,l6,l7,l8,l9,l10) -> Lext(p!3,n).Lext(p!2,n).Lext(p!1,n).Pit(s~e,l1!1,l2!2,l3!3,l4,l5,l6,l7,l8,l9,l10) kE DeleteMolecules
  Lext(p!4,n).Lext(p!3,n).Lext(p!2,n).Lext(p!1,n).Pit(s~p,l1!1,l2!2,l3!3,l4!4,l5,l6,l7,l8,l9,l10) -> Lext(p!4,n).Lext(p!3,n).Lext(p!2,n).Lext(p!1,n).Pit(s~e,l1!1,l2!2,l3!3,l4!4,l5,l6,l7,l8,l9,l10) kE DeleteMolecules
  Lext(p!5,n).Lext(p!4,n).Lext(p!3,n).Lext(p!2,n).Lext(p!1,n).Pit(s~p,l1!1,l2!2,l3!3,l4!4,l5!5,l6,l7,l8,l9,l10) -> Lext(p!5,n).Lext(p!4,n).Lext(p!3,n).Lext(p!2,n).Lext(p!1,n).Pit(s~e,l1!1,l2!2,l3!3,l4!4,l5!5,l6,l7,l8,l9,l10) kE DeleteMolecules
  # 5 more endocytosis reactions needed
  # endosome degradation
  Pit(s~e,l1,l2,l3,l4,l5,l6,l7,l8,l9,l10) -> Trash dE
  # the following reactions hit combinatorial complexity, but would be OK
  # if it were possible to use the state of the reactant in the product
  #!! lysis of endosome with 1 lipoplex - 11 reactions
  #Lext(p!1,n~?).Pit(s~e,l1!1,l2,l3,l4,l5,l6,l7,l8,l9,l10) -> Lint(n) kL
  Lext(p!1,n~345).Pit(s~e,l1!1,l2,l3,l4,l5,l6,l7,l8,l9,l10) -> Lint(n~345) kL
  Lext(p!1,n~346).Pit(s~e,l1!1,l2,l3,l4,l5,l6,l7,l8,l9,l10) -> Lint(n~346) kL
  Lext(p!1,n~347).Pit(s~e,l1!1,l2,l3,l4,l5,l6,l7,l8,l9,l10) -> Lint(n~347) kL
  Lext(p!1,n~348).Pit(s~e,l1!1,l2,l3,l4,l5,l6,l7,l8,l9,l10) -> Lint(n~348) kL
  Lext(p!1,n~349).Pit(s~e,l1!1,l2,l3,l4,l5,l6,l7,l8,l9,l10) -> Lint(n~349) kL
  Lext(p!1,n~350).Pit(s~e,l1!1,l2,l3,l4,l5,l6,l7,l8,l9,l10) -> Lint(n~350) kL
  Lext(p!1,n~351).Pit(s~e,l1!1,l2,l3,l4,l5,l6,l7,l8,l9,l10) -> Lint(n~351) kL
  Lext(p!1,n~352).Pit(s~e,l1!1,l2,l3,l4,l5,l6,l7,l8,l9,l10) -> Lint(n~352) kL
  Lext(p!1,n~353).Pit(s~e,l1!1,l2,l3,l4,l5,l6,l7,l8,l9,l10) -> Lint(n~353) kL
  Lext(p!1,n~354).Pit(s~e,l1!1,l2,l3,l4,l5,l6,l7,l8,l9,l10) -> Lint(n~354) kL
  Lext(p!1,n~355).Pit(s~e,l1!1,l2,l3,l4,l5,l6,l7,l8,l9,l10) -> Lint(n~355) kL
  #!! lysis of endosome with 2 lipoplexes - 11^2 reactions
  Lext(p!2,n~345).Lext(p!1,n~345).Pit(s~e,l1!1,l2!2,l3,l4,l5,l6,l7,l8,l9,l10) -> Lint(n~345) + Lint(n~345) kL
  # lysis of endosome with 10 lipoplexes - 11^10 reactions
  # lipoplex degradation
  Lint -> Trash dL
  #!! unpacking of lipoplex - 11 reactions with sum of 345 to 355 elements
  # Lint(n) -> n*mRNA kU
  # Lint(n~345) -> 345*mRNA kU, example with only 5*mRNA
  # here we could use the same method that we used in the SPiM model
  Lint(n~345) -> mRNA+mRNA+mRNA+mRNA+mRNA kU
  Lint(n~346) -> mRNA+mRNA+mRNA+mRNA+mRNA kU
  # mRNA degradation
  mRNA -> Trash dM
  # translation
  mRNA -> mRNA + GFP kTL
  # GFP degradation
  GFP -> Trash dG
  I -> I + Timer 1
end reaction rules

simulate_nf({suffix=>nf,t_end=>30*3600,n_steps=>300});
#writeXML();
```

## References

1. Varga CM, Tedford NC, Thomas M, Klibanov AM, Griffith LG, et al. (2005) Quantitative comparison of polyethylenimine formulations and adenoviral vectors in terms of intracellular gene delivery processes. Gene Ther 12: 1023-1032.

2. Zhou J, Yockman J, Kim S, Kern S (2007) Intracellular Kinetics of Non-Viral Gene Delivery Using Polyethylenimine Carriers. Pharmaceutical Research 24: 1079-1087.

3. Dinh AT, Pangarkar C, Theofanous T, Mitragotri S (2007) Understanding intracellular transport processes pertinent to synthetic gene delivery via stochastic simulations and sensitivity analyses. Biophys J 92: 831-846.

4. Kremers G-J, Goedhart J, van den Heuvel DJ, Gerritsen HC, Gadella TWJ (2007) Improved Green and Blue Fluorescent Proteins for Expression in Bacteria and Mammalian Cells. Biochemistry 46: 3775-3783.

5. Megerle JA, Fritz G, Gerland U, Jung K, Rädler JO (2008) Timing and Dynamics of Single Cell Gene Expression in the Arabinose Utilization System. Biophys J 95: 2103-2115.

6. Reid BG, Flynn GC (1997) Chromophore Formation in Green Fluorescent Protein. Biochemistry 36: 6786-6791.

7. Sniegowski JA, Lappe JW, Patel HN, Huffman HA, Wachter RM (2005) Base catalysis of chromophore formation in Arg96 and Glu222 variants of green fluorescent protein. Journal of Biological Chemistry 280: 26248-26255.

8. Tsien RY (1998) The green fluorescent protein. Annu Rev Biochem 67: 509-544.

9. Sneddon MW, Faeder JR, Emonet T (2011) Efficient modeling, simulation and coarse-graining of biological complexity with NFsim. Nat Meth 8: 177-183.
